# How has research on the effectiveness and safety of COVID-19 vaccination been evaluated: a scope review with emphasis on CoronaVac

**DOI:** 10.3389/fpubh.2024.1321327

**Published:** 2024-04-10

**Authors:** Juan C. Alzate-Ángel, Paula A. Avilés-Vergara, David Arango-Londoño, Alberto Concha-Eastman, Anthony Garcés-Hurtado, Liliana López-Carvajal, Ingrid L. Minotta, Delia Ortega-Lenis, Geraldine Quintero, Sebastián Reina-Bolaños, Carlos A. Reina-Bolaños, Pablo Roa, Melanie Sánchez-Orozco, Catalina Tovar-Acero, María P. Arbeláez-Montoya

**Affiliations:** ^1^Grupo de Epidemiología, Universidad de Antioquia, Medellín, Colombia; ^2^Grupo de Enfermedades Tropicales y Resistencia Bacteriana, Universidad del Sinú, Montería, Colombia; ^3^Grupo de investigación EMAP - Estadística y Matemáticas Aplicadas, Pontificia Universidad Javeriana, Cali, Colombia; ^4^Grupo de Investigación, Secretaría de Salud Distrital, Cali, Colombia; ^5^Grupo de Investigación Clínica - PECET (GIC-PECET), Universidad de Antioquia, Medellín, Colombia; ^6^Grupo de Investigación en Economía, Gestión y Salud, ECGESA. Pontificia Universidad Javeriana, Cali, Colombia; ^7^Departamento de Salud pública y Epidemiología, Pontificia Universidad Javeriana, Cali, Colombia

**Keywords:** COVID-19, SARS-CoV-2, vaccines, CoronaVac, review

## Abstract

**Introduction:**

The control of the COVID-19 epidemic has been focused on the development of vaccines against SARS-CoV-2. All developed vaccines have reported safety and efficacy results in preventing infection and its consequences, although the quality of evidence varies depending on the vaccine considered. Different methodological designs have been used for their evaluation, which can influence our understanding of the effects of these interventions. CoronaVac is an inactivated vaccine, and it has been assessed in various studies, including clinical trials and observational studies. Given these differences, our objective was to explore the published information to answer the question: how has the efficacy/effectiveness and safety of CoronaVac been evaluated in different studies? This is to identify potential gaps and challenges to be addressed in understanding its effect.

**Methods:**

A scoping review was carried out following the methodology proposed by the Joanna Briggs Institute, which included studies carried out in humans as of 2020, corresponding to systematic reviews, clinical trials, analytical or descriptive observational studies, in which the effectiveness and/or safety of vaccines for COVID19 were evaluated or described. There were no age restrictions for the study participants.

**Results:**

The efficacy/effectiveness and safety of this vaccine was assessed through 113 studies. Nineteen corresponded to experimental studies, 7 of Phase II, 5 of Phase IV, and 4 were clinical trials with random assignment. Although some clinical trials with random assignment have been carried out, these have limitations in terms of feasibility, follow-up times, and with this, the possibility of evaluating safety outcomes that occur with low frequencies. Not all studies have used homogeneous methods of analysis. Both the prevention of infection, and the prevention of outcomes such as hospitalization or death, have been valued through similar outcomes, but some through multivariate analysis of dependencies, and others through analysis that try to infer causally through different control methods of confounding.

**Conclusion:**

Published information on the evaluation of the efficacy/effectiveness and safety of the CoronaVac is abundant. However, there are differences in terms of vaccine application schedules, population definition, outcomes evaluated, follow-up times, and safety assessment, as well as non-standardization in the reporting of results, which may hinder the generalizability of the findings. It is important to generate meetings and consensus strategies for the methods and reporting of this type of studies, which will allow to reduce the heterogeneity in their presentation and a better understanding of the effect of these vaccines.

## 1 Introduction

Starting from the first reports coming from China and from countries in Europe and Asia, about the infection produced by SARS-CoV-2, its high contagion, and lethality of up to 14% in older adults, and the subsequent declaration of a COVID-19 pandemic, and together with the measures established by the healthcare authorities to manage the disease, efforts began to develop effective and safe vaccines that would contribute to speeding up the control of this health condition, through the reduction of infections, complications, and deaths associated with this disease (1).

For this reason, pandemic control efforts have focused on developing vaccines against SARS-CoV-2 that are capable of acting against infection, disease, or transmission, and thus contribute to disease control (2). In this context, different research groups have developed vaccines using different platforms, including mRNA, viral vectors, and inactivated viruses (3).

Unlike most drugs, whose benefits are limited to the individual taking them, vaccines have the potential to produce far-reaching effects on general public health and well-being, cognitive development, and, ultimately, economic productivity (4). However, the global advances in vaccination coverage achieved during the first years of the 21st century have been threatened by the emergence of anti-vaccination groups that have questioned vaccine efficacy to create public distrust of vaccines and immunization programs. This requires an adequate and conscious evaluation of both the efficacy/effectiveness and the different aspects that can affect the safety of the people who receive them (5).

In general, vaccines that have gained approval for human use have been effective in preventing COVID-19, particularly in preventing severe disease and death. However, reports on their implementation are mainly based on follow-up studies of the adult population (6). Additionally, if the vaccination prevents symptoms from developing and asymptomatic infections are less likely to be discovered than symptomatic ones, it is feasible that the effectiveness against any infection has been overstated. A competitive tendency toward underestimate arises when estimates are based on tests with inadequate specificity, particularly when testing are conducted more frequently than has been estimated for various COVID-19 vaccinations (7).

All vaccines seem to be safe and efficacious against all variations of interest in preventing hospitalization, death, and severe COVID-19; however, the quality of the data differs significantly between the vaccines under consideration (8).

Different methodological designs have been used to evaluate the effectiveness and safety of vaccines for COVID-19. Most clinical trials were carried out before the appearance of variants of concern, and the duration, subgroups evaluated, and analysis methods were not homogeneous between vaccines, creating uncertainty about some effects and comparisons (9).

CoronaVac is an inactivated whole-virus vaccine against COVID-19 adjuvanted with aluminum hydroxide created from African green monkey kidney cells (Vero cells) inoculated with SARS-CoV-2 (strain CN02). The Chinese company Sinovac Biotech developed the vaccine, and on June 1, 2021, the World Health Organization (WHO) approved the vaccine for emergency use (10). Using two 3 μg doses of CoronaVac, the overall efficacy for avoiding symptomatic COVID-19 (before the emergence of concerning variations) has been assessed at 67.7% (95%CI: 35.9 to 83.7%) (10). Compared to COVID-19 prevention, its impact in preventing hospital stays, ICU admissions, and fatalities has been much stronger. Three-dose regimens have also been shown to raise seroconversion levels of neutralizing antibodies, even against variants like Omicron. Few serious vaccine-related adverse reactions have been reported (10).

However, given the differences that may exist in the methods used to assess the efficacy, effectiveness, and safety of vaccines against COVID-19, our objective was to explore the published research on COVID-19 vaccines, focusing on CoronaVac, in order to answer the question: How has the efficacy/effectiveness and safety of CoronaVac been assessed in different designs and study phases of the vaccines used to control COVID-19?

## 2 Methods

A scoping review was carried out under a protocol registered in the Open Science Framework (OSF; osf.io/aeut4), and following the methodology proposed by the Joanna Briggs Institute (11), which included studies carried out in humans as of 2020, corresponding to systematic reviews, clinical trials, and analytical or descriptive observational studies in which the effectiveness and/or safety of vaccines for COVID19 were evaluated or described. There were no age restrictions on the study participants.

Abstracts from congresses were not evaluated because they had not been subjected to systematic peer evaluation at the time, nor were studies published in languages other than English or Spanish.

### 2.1 Search methods for study identification

To identify potentially relevant articles for review, the following databases were searched, starting from 2020: MEDLINE, EMBASE, LILACS, Scopus, and Cochrane.

The following valid strategy was used for MEDLINE through PubMed and then adapted to other databases:

(((SARS-CoV-2[MeSH Terms]) OR (COVID-19[MeSH Terms])) OR (Coronavirus[MeSH Terms])) AND ((COVID-19 Vaccines[MeSH Terms]) OR (Coronavirus vaccines[Title/Abstract])).

The full search strategy is presented in the Supplementary material.

### 2.2 Study selection

The initial screening of the studies was independently performed by two reviewers in pairs (PA-AG and PR-SR). The RIS files of each database were uploaded to Rayyan software (12). Disagreements were resolved by a third author (JA).

Both reviewers assessed all titles and abstracts and excluded those considered irrelevant for the review, those not meeting the inclusion criteria, or because they were duplicates. Subsequently, 15 reviewers independently (JA, PA, DA, AC, AG, LL, LM, DO, GQ, SR, CR, PR, MS, CT, MA) evaluated the full text of the studies to verify the eligibility criteria. A cross-review was carried out for studies evaluating CoronaVac by four reviewers (PA, AG, PR, and SR).

### 2.3 Variable

Of the definitively selected studies, the following variables were extracted in a paired form: (i) type of study, (ii) population studied, (iii) intervention (vaccine) evaluated, (iv) control, (v) follow-up time, (vi) efficacy and/or effectiveness outcomes, and (vii) safety outcomes.

### 2.4 Data synthesis

For each outcome, a description of the results was made following the description in the document and/or Supplementary material of the article.

## 3 Results

### 3.1 Study selection

**Figure 1:**
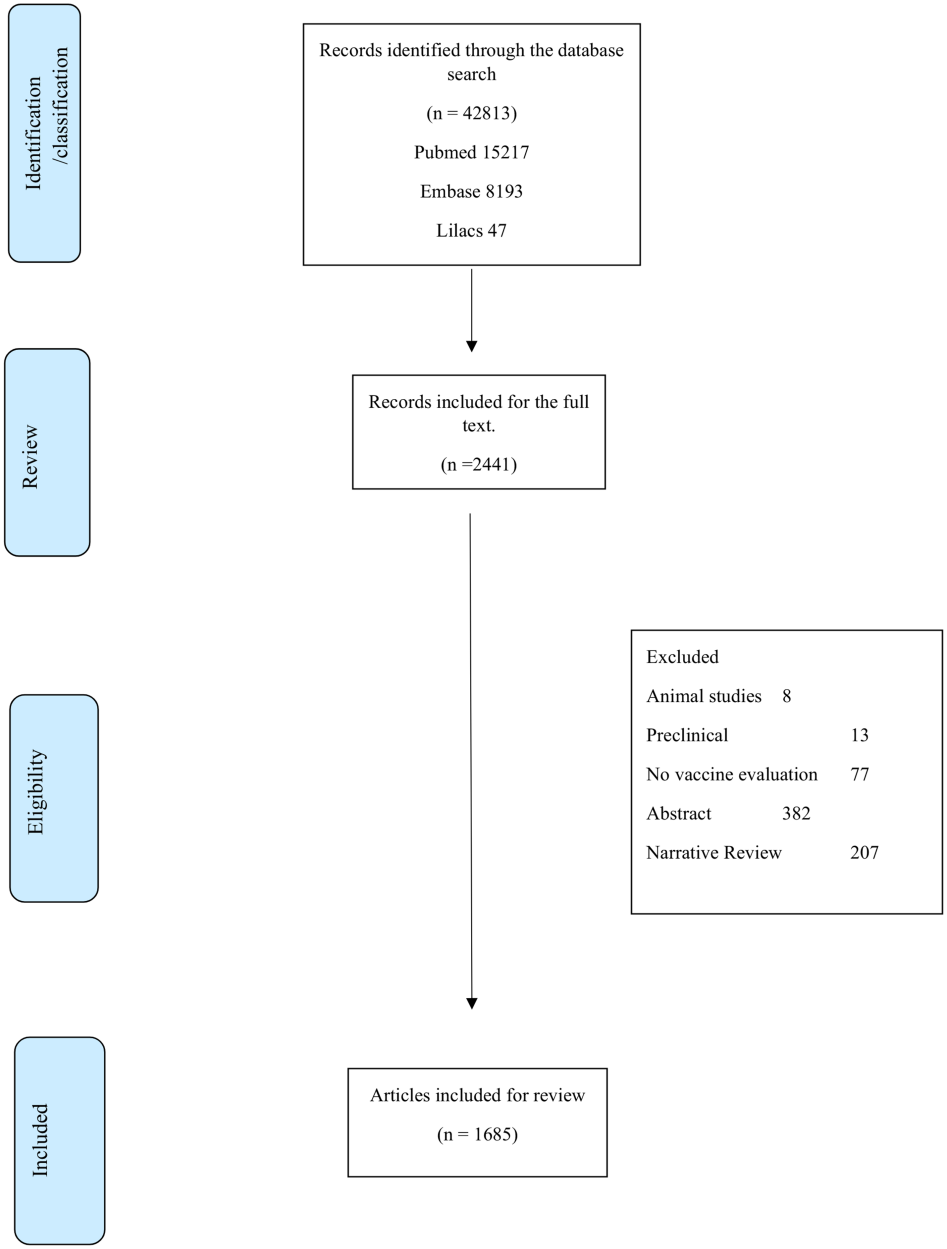
Flow diagram of the literature review process.

The search identified 42,813 titles for the initial evaluation, of which 40,372 were excluded after a review of the title, abstract, and possible duplication. A total of 2,441 full texts were reviewed to verify the eligibility criteria, of which 1,685 were included in the synthesis (Figure 1; Supplementary material).

### 3.2 Synthesis of the results

One hundred vaccines were evaluated through randomized clinical trials (RCT). The other studies corresponded to observational studies, 705 (43.9%) analytical studies, and mainly cohort studies (467; 29.1%). Three hundred and seventy-seven patients (23.5%) were series or case reports.

One hundred twenty-six studies (7.8%) did not specify the vaccine evaluated. Other studies have evaluated one or more specific vaccines. Seven hundred thirty-two studies did not include a vaccine or a control group. Two hundred and thirty-eight evaluated several types of vaccines, and 160 compared a vaccine against a placebo. The number of patients or vaccine doses evaluated in each study went from one (case report) to 306,473,169 doses of applied vaccines (13).

Regarding the population assessed, 44.4% of the studies evaluated the effects of vaccines on adults. 3.4% in adults and adolescents, 2% in adolescents, 1.2% in immunosuppressed individuals, 1.2% in children, 0.9% in pregnant women, and 0.25% in people living with HIV. The overall monitoring time ranged from hours to 6 months; this difference occurred between studies that evaluated immunological outcomes, which could occur within hours or days, and those that evaluated clinical outcomes.

A total of 15.1% of the studies evaluated the effectiveness or efficacy of vaccines by evaluating their effects on preventing infection, hospitalization, or death from infection. 59.1% of the studies corresponded to the description of safety events. The events were described heterogeneously. In some studies, they are only recorded as “mild adverse events” or “mild systemic events.” Few studies reported specific events such as myocarditis, and hepatic or allergic alterations. Of the studies, 25.8% described immunological outcomes, 368 studies through the measurement of antibodies, and 64 through the effects mediated by cellular immunity.

#### 3.2.1 CoronaVac

The efficacy, effectiveness, and safety of this vaccine have been assessed in 113 studies. Nineteen corresponded to experimental studies, seven of Phase II, five of Phase IV, and four were clinical trials with random assignment, carried out in adults in Chile, Indonesia, and Turkey (14–17), comparing the effect of the vaccine versus placebo. The other studies were observational studies, most of which were case reports, case series, or descriptions of cohorts. Of these, 45.1% were conducted in Asia, 23% in Latin America, and 22.1% in Europe, mainly in Turkey (of 27/29 European studies).

As for the population, 87.6% of the studies were conducted in adults, while the representation of studies in pregnant women, children, immunosuppressed people, or people living with HIV ranged between 0.9 and 3.5% of the studies.

Sixty studies (53.1%) evaluated the effect of CoronaVac in a control group. The others were case reports or descriptions of cohorts without comparison. Of these, 42 (70%) described events in patients who received CoronaVac and another vaccine, without performing an effectiveness or efficacy analysis. Other studies evaluated the efficacy and effectiveness by measuring the effect of preventing hospitalization, death, or COVID. Of the total, 34 studies evaluated CoronaVac (30.1%) and described some immune outcomes.

Although the objective of the review was not to assess the effectiveness of the vaccine, but rather how it has been evaluated, the results of some of the identified studies are shown below in order to present relevant information about the methods used and possible differences between them, which lead to discussing the effect that this can have on the analysis and use of CoronaVac and other vaccines. More details on the results of the identified studies can be found in the Supplementary material section.

#### 3.2.2 Efficacy/effectiveness of CoronaVac

##### 3.2.2.1 Prevention of COVID-19

Cheng et al. (18) evaluated the effectiveness of BNT162B2 and CoronaVac in patients with chronic kidney disease in Hong Kong. 28,374 people were not vaccinated, 27,129 received two doses of BNT162b2, and 47,640 received two doses of CoronaVac in this retrospective cohort analysis. Following inverse probability of treatment weighting with 1% extreme values, a cohort that was well-balanced and had a standardized mean difference of less than 0.1 was generated.

The effectiveness of CoronaVac on Turkish healthcare professionals was assessed by Can et al. (19). 4,067 medical personnel worked at a University Hospital in Istanbul, where this retrospective cohort study was carried out. In the fully vaccinated group, the follow-up period was defined as beginning 14 days following the second dose. If PCR test findings were positive or the trial came to an end, healthcare personnel were excluded. Healthcare personnel who were not vaccinated were prohibited from participating in any COVID-19 vaccination. The vaccine’s unadjusted and adjusted effectiveness were calculated using the incidence rate ratio and Cox regression. 29% of the healthcare staff had not received any vaccinations, whereas 71% had received all recommended doses.

Jara et al. (20) conducted an evaluation of a prospective, observational, national-level cohort of individuals (≥ 16 years) associated with the Fondo Nacional de Salud insurance program in Chile. They used individual-level data to assess the efficacy of booster vaccines, namely BNT162B2 (Pfizer-Biontech), AZD1222 (Oxford-AstraZeneca), and CoronaVac (Syovac Biotech), in individuals who had completed a primary immunization schedule with CoronaVac, in comparison to those who had not received any vaccinations. The hazard ratios were estimated using inverse probability-weighted and stratified survival regression models that took into account the time-varying vaccination status and adjusted for pertinent clinical, socioeconomic, and demographic confounders. An estimate was made of the change in risk associated with the primary immunization series and booster shot from being unvaccinated to vaccinated. 11,174,257 persons in total fulfilled the trial’s eligibility conditions; of these, 4,127,546 finished the two doses of the CoronaVac primary immunization regimen and got a booster dose during the study period. 2,019,260 (48.9%) individuals received a BNT162b2 booster, 186,946 (4.5%) received a homologous booster with CoronaVac, and 1,921,340 (46–5%) participants received an AZD1222 booster. The weighted stratified Cox model was utilized to compute the modified vaccination efficacy in preventing COVID-19.

Utilizing hospitalization, vaccination, and National COVID-19 notification data, Cerqueira-Silva et al. (21) conducted a case–control study in Brazil to evaluate the efficacy of four vaccines (CoronaVac [synovac], ChAdOx1 nCoV-19 [AstraZeneca], Ad26.COV2.S [Janssen], and BNT162b2 [Pfizer-Bionntech]) in individuals with laboratory-confirmed prior SARS-COV-2 infection. The probabilities of test positivity and the likelihood of hospitalization or death from COVID-19 were compared based on vaccination status and the amount of time that had passed from the first or second dose of vaccinations using multivariable conditional logistic regression.

The same authors conducted a similar study in Brazil (22), using linked national Brazilian databases to conduct a negative-test design study with nearly 14 million participants (~ 16 million tests) to estimate the effectiveness of the CoronaVac vaccine over time and the BNT162B2 booster vaccination against severe COVID-19 outcomes (hospitalization or death) and severe acute respiratory syndrome, as confirmed by RT-PCR (SARS-COV-2).

To evaluate the effectiveness of homologous and heterologous boosters against COVID-19 in the context of OMICRON, Ranzani et al. (23) conducted a nationwide case–control study (with negative PCR results) to assess homologous and heterologous (BNT162B2) booster doses in adults who received two doses of CoronaVac in Brazil in the OMICRON context.

A case–control research was carried out in Thailand by Sritipsukho et al. (24) to assess the efficacy of various vaccination regimens in preventing COVID-19 during the time when the delta variant was the predominant causing virus (≥ 95%). By correcting for individual demographic and clinical factors, the efficacy of vaccines was assessed.

##### 3.2.2.2 Prevention of hospitalization and death

Cheng et al. (18) found that both vaccines reduced hospitalization and death related to COVID-19, which was the opposite of the outcome of preventing COVID-19 infection. The vaccination efficacy for BNT162b2 users was 64% (95% CI: 57–69%) for hospitalization associated to COVID-19 and 86% (95% CI: 80–90%) for COVID-19-related death. Regarding hospitalization and death associated to COVID-19, the vaccine efficacy for CoronaVac was 44% (95% CI: 37–49%) and 70% (95% CI: 64–75%), respectively.

In the Jara et al. (20) study, the adjusted effectiveness of the vaccine against hospitalization due to COVID-19, ICU admission, and death was 86.3% (83.7–88.5), 92.2% (88.7–94.6), and 86.7% (80.5–91.0) for a CoronaVac homolog booster; 96.1% (95.3–96.9), 96, 2% (94.6–97.3), and 96.8% (93.9–98.3) for a BNT162b2 booster; and 97.7% (97.3–98.0), 98.9% (98.5–99.2), and 98.1% (97.3–98.6) for an AZD1222 booster, respectively.

In Brazil (21), the effectiveness against hospitalization or death 14 or more days after the completion of the vaccination schedule was 81.3% (75.3–85.8) for CoronaVac, 89.9% (83.5–93.8) for ChAdOx1 nCoV-19, and 57.7% (−2.6–82.5) for Ad26.COV2.S, and 89.7% (54.3–97.7) for BNT162b2.

##### 3.2.2.3 Immunological outcomes

Bueno et al. (14), conducting a randomized placebo-controlled clinical trial in Chile, assessed the effectiveness of CoronaVac by assigning participants to either a placebo or two doses of CoronaVac spaced 2 weeks apart. Enrollments totaled 434, with 397 individuals in the 18–59 age range and 37 in the 60+ age range. 81 subjects had hemoral assessments. 2 and 4 weeks after the second dosage, respectively, the seroconversion rates for specific anti-S1-receptor binding domain (RBD) immunoglobulin G (IgG) were 82.22 and 84.44% in the 18–59 years age group and 62, 69 and 70.37% in the ≥60 years age group. A notable rise in the amount of neutralizing antibodies in circulation was noted two and 4 weeks following the second dosage. 47 participants had their cells evaluated. After stimulation with Mega Pools of SARS-CoV-2 peptides, a notable increase in T cell responses was seen, as evidenced by the release of interferon-γ (IFN-γ).

According to Zeng et al. (25) the following were the findings of two single-center, double-blind, randomized, placebo-controlled phase II clinical trials: adults from Jiangsu, China, aged 18 to 59 years were first assigned (1:1) into two vaccination schedule cohorts: one for the days 0 and 14 of vaccination (cohort 1), and another for the days 0 and 28 of vaccination (cohort 2). Each cohort was then randomly assigned (2:2:1) to either a placebo group or a 3 μg or 6 μg dose of CoronaVac. A third dose was given to half of the participants in each cohort 6 months after the second dose, and an additional dose was given to the other half of the individuals 28 days following the second dose, as a result of a protocol revision. In a separate phase II experiment carried out in Hebei, China, individuals who met the eligibility criteria of 60 years or above were randomized to receive three injections of 1.5, 3, or 6 μg of vaccine or a placebo. The first two doses of the vaccine were given 28 days apart, while the second and third doses were given 6 months apart. For the per-protocol population (those who finished their allotted third dose), the primary research outcomes were geometric mean titers (GMTs), geometric mean increments (GMIs), and seropositivity of neutralizing antibodies to SARS-CoV-2. Out of the 600 participants, who were between the ages of 18 and59, 540 (90%) were qualified for a third dose. Of these, 269 (50%) received the third primary dose (cohorts 1a-14d-2 m and 2a-28d-2 m) 2 months after the second dose, and 271 (50%) received a booster dose 8 months later (cohorts 1b-14d-8 m and 2b-28d-8 m). For the 1b-14d-8 m cohort (*n* = 53; GMT 3.9 [95% CI 3.1–5.0]) and 2b-28d-8 m cohort (*n* = 49; GMT 6.8 [95% CI 5.2–8.8]), neutralizing antibody titers elicited by the first two treatments in the 3 μg group declined after 6 months to close or below the seropositive cut-off point (GMT of 8). The GMTs measured 14 days later increased to 137.9 (95% CI: 99.9–190.4) for the 1b-14d-8 m cohort and to 143.1 (110.8–184.7) 28 days later for the 2b-28d-8 m cohort when a booster dose was administered 8 months following a second dose. After the principal third dosage, GMTs increased somewhat in cohorts 1a-14d-2 m (n = 54) and 2a-28d-2 m (*n* = 53). In cohort 1a, GMTs increased from 21.8 (95% CI: 17.3–27.6) on day 28 after the second dose to 45.8 (35.7–58.9) on day 28 after the third dose. Six months following the third dose, GMTs had dropped to almost the positive threshold: in the 1a-14d-2 m group, they were 9.2 (95% CI 7.1–12.0), while in the 2a-28d-2 m cohort, they were 10.0 (7.3–13.7). Similarly, 6 months following the initial two-dose series, neutralizing antibody titers dropped to almost or below the seropositive threshold among people 60 years of age or older who received booster doses (303 [87%] of 350 participants were eligible for a third dosage). Eight months following the second treatment, which markedly raised neutralizing antibody concentrations, a third dose was administered: After the second dose on day 28, GMTs climbed to 42.9 (95% CI: 31.0–59.4), and after the third dose on day 28 (*n* = 29), GMTs increased to 158.5 (96.6–259.2).

Chantasrisawad et al. (26) assessed healthy children aged 5 to 11 who were given two intramuscular doses of either Covilo or CoronaVac and 10 μg of BNT162b2. Neutralizing antibodies against the Omicron version were assessed using a pseudovirus neutralization test (pVNT, ID50) and a surrogate viral neutralization test (sVNT, % inhibition) 14–21 days following the booster. The antibody responses were contrasted with those of a concurrent cohort of kids who got two BNT162b2 doses separated by 3 weeks. A total of 59 children, consisting of 20 CoronaVac recipients and 39 Covilo recipients, were registered between April and May 2022, with a mean age (SD) of 8.5 years (1.7). The primary series’ median interval was 49 days, with an interquartile range of 33–51. Following the booster, the geometric means (MG) of pVNT and sVNT were 499 (95%CI: 399–624) and 72.2% inhibition (95%CI: 67.2–77.6), respectively. From zero to 72 %, the percentage of kids with sVNT against Omicron strain ≥68% inhibition rose. In comparison to the parallel cohort, the geometric mean ratios (GMR) of sVNT and pVNT were 4.3 and 12.2, respectively. In comparison to children who received a booster dosage between 4 and 6 weeks, the GMR of sVNT and pVNT among those who received it at a time interval of more than 6 weeks was 1.2 (95% CI: 1.1–1.3) and 1.8 (95% CI 1.2–2.7).

In Turkey, (27) et al. assessed the variables influencing the antibody response in 235 adults over 65 years of age following two doses of the inactivated SARS-CoV-2 vaccination (CoronaVac). Four weeks following the first and second vaccination doses, the mean levels of anti-SARS-CoV-2 IgG antibodies were 37.70 ± 57.08 IU/mL and 194.61 ± 174.88 IU/mL, respectively. Additionally, 4 weeks following the first vaccination dose, 134 out of 235 participants (57.02%) had an antibody level of less than 25.6 IU/mL (negative); 4 weeks following the second vaccination dose, this percentage was 11.48% (*n* = 27). Eight participants (29.6%) had no comorbidities, while 19 (70.4%) with an antibody level less than 25.6 IU/mL 4 weeks after the first dose of the vaccination had at least one comorbid condition, including diabetes mellitus (*F* = 2.352, *p* = 0.006). Individuals with comorbidities and those 65 years of age or older showed lower antibody response rates.

Demirbakan et al. (28) examined the presence of immunoglobulin G antibodies in the receptor-binding region of the S1 subunit of the SARS-CoV-2 spike protein in 1072 healthcare workers following immunization in a descriptive observational research. 28 days, 21 days, and 3 months following the first, second, and second dosages, respectively, were the times at which blood samples were taken. Anti-spike antibodies were found in 834/1072 (77.8%) subjects 4 weeks following the initial vaccination dose. Between 18 and 34 years of age, seropositivity was observed to be greater in both men and women (84.6%) compared to 70.6% (*p* < 0.001) in the former group. In 1008 of 1,012 (99.6%) cases, anti-spike antibodies were found 21 days after the second dose, and in 803 of 836 (96.1%) cases, anti-spike antibodies were found 3 months later.

##### 3.2.2.4 Safety

According to Bueno et al. (14) in their placebo-controlled clinical trial, pain at the injection site was the primary adverse reaction in 434 volunteers, and it occurred more frequently in the vaccine arm than in the placebo arm. The majority of the negative effects that were seen were modest and limited. No significant negative events were noted.

The frequency of adverse reactions was reported by Zeng et al. (25) without providing any additional effect measurements. In every immunization group, all adverse responses that were reported within 28 days after the third dose were classified as either grade 1 or 2. In the 1a-14d-2 m cohort, 150 participants reported three serious adverse events (2%); in the 1b-14d-8 m cohort, 150 participants reported four (3%); in the 2a-28d-2 m and 2b-28d-8 m cohorts, 150 participants reported one (1%); overall 349 people reported 24 (7%) serious adverse events.

Cheng et al. (18) observed an incidence rate of any adverse events of special interest following the first vaccination dose of 34.28 (95% CI: 29.81–39.23) and 38.39 (95% CI: 34.81–42.23) per 10,000 doses of BNT162b2 and CoronaVac, respectively, in their retrospective cohort of patients with chronic kidney disease. BNT162b2 (incidence rate ratio [95% CI]: first dose: 0.86 [0.69–1.08]; second dose: 0.96 [0.76–1.22]; third dose: 0.60 [0.33–1.10]) and CoronaVac (incidence rate ratio [95% CI]: first dose: 0.76 [0.64–0.91]; second dose: 0.86 [0.71–1.05]; third dose: 0.74 [0.36–1.54]) did not show an increased risk of overall adverse event of special interest when compared to the baseline period.

## 4 Discussion

The COVID-19 pandemic has affected the world’s population with a high morbidity and mortality rate. Recent reports have described persistent symptoms that extend beyond the initial period of the disease. It has been observed that adverse consequences, in addition to respiratory effects, are produced at different levels: cardiovascular, neurological, or immunological; cutaneous, gastrointestinal, or kidney manifestations, as well as in mental health, both as a result of acute infection and by the so-called post-COVID-19 syndrome (29). In this context, developing effective and safe vaccines was the determining control measure for pandemic management since, in addition to reducing the transmission of infections and allowing the control of the disease, vaccines had a determining role in reducing severe and fatal complications associated with infection (30). In addition to the above, the time in which the vaccine candidates were available, where it took less than a year for developers to complete the design, manufacturing, efficacy and safety testing and evaluation and approval for use, is an immeasurable scientific and public health learning, as well as an example of cooperation between healthcare authorities, the scientific community and private sector (31).

This review presents an analysis of the methods, populations, and scope of the studies that have evaluated the efficacy/effectiveness and safety of the vaccines available for COVID-19, emphasizing CoronaVac. Differences were found in terms of the proportion of populations evaluated, follow-up times, and times of the studies regarding the appearance of variants of concern.

Although some clinical trials with random assignment have been carried out to assess efficiency and safety outcomes with CoronaVac, these have limitations in terms of feasibility, follow-up times, and with this, the possibility of evaluating safety outcomes that occur with low frequencies (32). In this sense, it is important to carry out observational data analysis. However, not all studies have used homogeneous methods of analysis. Both the prevention of infection, and the prevention of outcomes such as hospitalization or death, have been valued through similar outcomes, but some through multivariate analysis of dependencies, and others through analysis that try to infer causally through different control methods of confounding. Studies have compared the evaluation of the same outcome through different methods, including multivariable logistic regression, propensity matching, propensity adjustment, and propensity-base weighting. However, researchers described that the estimates are very sensitive to the explicit or implicit weighting system in an adjustment technique, so it must be clear for which population a global treatment estimate is most appropriate (33).

It is important to recognize that there are common challenges in the collection, notification, and use of epidemiological data, such as the exhaustiveness and representativeness of the results and their comparability in time, among others. Therefore, it is necessary to identify the strongest analytical designs (among them the interrupted temporal series and comparative longitudinal studies), accompanied by sensitivity analysis of the results and being explicit, starting from the design, in the type of biases and problems that can be found in the data analysis that is available (34).

Concerning the evaluation of the immune response to the different types of vaccines, it has been oriented both to the antibody-mediated response and that mediated by cellular immunity. Among the antibody-mediated response, the reference standard has been established with the specific neutralizing antibody response against spike proteins of the virus, and a proxy to this response assessing neutralizing capacity has been measured in other studies by immunoglobulin G (IgG) antibody levels against the SARS-CoV-2 receptor binding domain (RBD) (35).

In the different studies, the decrease in the response levels to specific neutralizing antibodies was assumed to indicate the vaccine protection level when the levels of specific neutralizing antibodies fell between 4 and 6 months. The statistical methods used for their measurement are not homogeneous among all studies which has been used to recommend the application of boosters with vaccines produced in homologous or heterologous platforms of those received in established vaccination schemes (36, 37).

To assess the duration of vaccine protection in the real world, it is also important to consider the difficulties in assessing the cellular memory immune response. The measurement of the CD4+ and CD8+ T lymphocytes response expressed in the production of different activation markers is heterogeneous, depending on antigenic stimuli such as peptides from circulating virus variants, cells from infected individuals, or peptides from different vaccines, in addition to diversity in the host response, which does not allow to have precise indicators to define optimal vaccination schedules (38, 39).

In this context, inactivated whole virus vaccines, such as CoronaVac, by preserving epitopes of the virus, could respond in a broader spectrum to the different variants of circulating viruses or to new mutations, which could lead to the optimization of global vaccination schedules (10).

The main strength of our study lies in its systematic development, which reduces the possibility of biases in study selection. The use of different databases, including Latin American ones, allows for a broader search, although it is acknowledged that due to the magnitude of research on this topic, there may still be unreported or unfound studies, behaving as gray literature. The review results enabled us to achieve our objective, which was to describe how the efficacy/effectiveness of COVID-19 vaccines has been evaluated, with emphasis on CoronaVac. This allowed for the identification of some differences in these methods and some persisting gaps in defining more homogeneous methods for evaluation, regardless of whether these studies had high or low certainty in their evidence, which should be revisited if the objective is to evaluate the effectiveness and/or safety in the population of these interventions. However, the findings presented could be assessed and discussed with broader groups of experts in the field, which would help generate more accurate recommendations regarding their significance and potential implications.

In addition to the mentioned limitations, it is important to acknowledge that this type of review, having less precise question definitions compared to systematic reviews of effectiveness and safety (with their PICO structure), may result in some gaps in the application of search terms that could affect the results. Additionally, the vast amount of information, as was the case in our review, can create difficulties in synthesis and analysis, so it is crucial, as mentioned, to continue the discussion in groups with increasingly greater expertise in the subject (40). Lastly, while it is tempting to provide quantitative results regarding the synthesis conducted, the most important aspect is to address the original question regarding the gaps in the evaluation of these vaccines.

## 5 Conclusion

Published information on the evaluation of the efficacy/effectiveness and safety of the different vaccines against COVID-19 is abundant. However, there are differences in terms of vaccine application schedules, population definition, outcomes evaluated, follow-up times, and safety assessment, as well as non-standardization in the reporting of results, which may hinder the generalizability of the findings. It is important to define the relevance of the analysis methods in advance, considering these differences and the heterogeneity that can be produced in the analysis and meta-analysis of this information. It is important to generate meetings and consensus strategies for the methods and reporting of this type of studies, which will allow to reduce the heterogeneity in their presentation and a better understanding of the effect of these vaccines.

## Data availability statement

The original contributions presented in the study are included in the article/Supplementary material, further inquiries can be directed to the corresponding author.

## Author contributions

JA-Á: Conceptualization, Methodology, Writing – original draft, Writing – review & editing, Investigation. PA-V: Writing – original draft, Writing – review & editing, Investigation. DA-L: Writing – original draft, Writing – review & editing, Investigation. AC-E: Writing – original draft, Writing – review & editing, Investigation. AG-H: Writing – original draft, Writing – review & editing, Investigation. LL-C: Writing – original draft, Writing – review & editing, Investigation. IM: Writing – original draft, Writing – review & editing, Investigation. DO-L: Writing – original draft, Writing – review & editing, Investigation. GQ: Writing – original draft, Writing – review & editing, Investigation. SR-B: Writing – original draft, Writing – review & editing, Investigation. CR-B: Funding acquisition, Writing – original draft, Writing – review & editing, Investigation. PR: Writing – original draft, Writing – review & editing, Investigation. MS-O: Writing – original draft, Writing – review & editing, Investigation. CT-A: Writing – original draft, Writing – review & editing, Investigation. MA-M: Funding acquisition, Project administration, Writing – original draft, Writing – review & editing, Investigation, Project Administration.
