# Supplementary material for: How has research on the effectiveness and safety of COVID-19 vaccination been evaluated: a scope review with emphasis on CoronaVac
Source: Front Public Health. 2024 Apr 10;12:1321327. doi: 10.3389/fpubh.2024.1321327 (PMC11040685; doi:10.3389/fpubh.2024.1321327)
Supplement: Supplementary file 1 [file Data_Sheet_1.docx]

**HOW HAS RESEARCH ON THE EFFECTIVENESS AND SAFETY OF COVID-19 VACCINATION BEEN EVALUATED: A SCOPE REVIEW WITH EMPHASIS ON CORONAVAC**

**Supplementary Material**

Database search

| **Characteristic** | **Report** | | |
| --- | --- | --- | --- |
| Type of Search | New | | |
| Database | MEDLINE | | |
| Platform | Pubmed | | |
| Date | 23/10/2022 | | |
| Date range search | 2020 - 2022 | | |
| Language restrictions | English-Spanish | | |
| Other limits | Humans | | |
| Search strategy | **Nº** | **Terms** | **Results** |
|  | 1 | SARS-CoV-2[MeSH Terms] | 138.749 |
|  | 2 | COVID-19[MeSH Terms] | 191.239 |
|  | 3 | Coronavirus[MeSH Terms] | 152.167 |
|  | 4 | #1 OR #2 OR #3 | 206.191 |
|  | 5 | COVID-19 Vaccines[MeSH Terms] | 16.380 |
|  | 6 | Coronavirus vaccines[Title/Abstract] | 160 |
|  | 7 | #5 OR #6 | 16.478 |
|  | 8 | #4 AND #7 | 15.784 |
|  | 9 | #8 AND Filters: Humans, English, Spanish, from 2020/1/1- 2022/10/23 | 15.217 |
| Identified references | 15.217 | | |

| **Characteristic** | **Report** | | |
| --- | --- | --- | --- |
| Type of Search | New | | |
| Database | Embase | | |
| Platform | Embase | | |
| Date | 23/10/2022 | | |
| Date range search | 2020 - 2022 | | |
| Language restrictions | English-Spanish | | |
| Other limits | Humans | | |
| Search strategy | **Nº** | **Terms** | **Results** |
|  | 1 | 'sars coronavirus'/exp OR 'sars coronavirus' | 15.093 |
|  | 2 | 'coronavirus disease 2019'/exp OR 'coronavirus disease 2019' | 271.943 |
|  | 3 | 'coronavirinae'/exp OR 'coronavirinae' | 107.729 |
|  | 4 | #1 OR #2 OR #3 | 313.800 |
|  | 5 | 'sars-cov-2 vaccine'/exp OR 'sars-cov-2 vaccine' | 26.345 |
|  | 6 | 'covid-19 vaccine'/exp OR 'covid-19 vaccine' | 27.924 |
|  | 7 | #5 OR #6 | 28.192 |
|  | 8 | #4 AND #7 | 24.682 |
|  | 9 | #8 AND (2020:py OR 2021:py OR 2022:py) AND [embase]/lim NOT ([embase]/lim AND [medline]/lim) AND 'human'/de | 8.193 |
| Identified references | 8.193 | | |

| **Characteristic** | **Report** | | |
| --- | --- | --- | --- |
| Type of Search | New | | |
| Database | Lilacs | | |
| Platform | Biblioteca Virtual en Salud | | |
| Date | 23/10/2022 | | |
| Date range search | 2020 - 2022 | | |
| Language restrictions | English-Spanish | | |
| Other limits | Humans | | |
| Search strategy | **Nº** | **Terms** | **Results** |
|  | 1 | (mh:(SARS-CoV-2)) | 139.800 |
|  | 2 | (mh:(COVID-19)) | 198.585 |
|  | 3 | (mh:(Coronavirus)) | 8.324 |
|  | 4 | #1 OR #2 OR #3 | 204.136 |
|  | 5 | (mh:(COVID-19 Vaccines)) | 2.430 |
|  | 6 | (mh:(vaccines)) | 28.160 |
|  | 7 | #5 OR #6 | 28.160 |
|  | 8 | #4 AND #7 | 2.493 |
|  | 9 | #8 AND Filters: Lilacs, Inglés, Español, from 2020-2022 | 47 |
| Identified references | 47 | | |

| **Characteristic** | **Report** | | |
| --- | --- | --- | --- |
| Type of Search | New | | |
| Database | Scopus | | |
| Platform | Scopus | | |
| Date | 23/10/2022 | | |
| Date range search | 2020 - 2022 | | |
| Language restrictions | English-Spanish | | |
| Other limits | Humans | | |
| Search strategy | **Nº** | **Terms** | **Results** |
|  | 1 | TITLE-ABS-KEY ( "SARS-Cov-2" ) | 165.937 |
|  | 2 | TITLE-ABS-KEY ( "COVID-19" ) | 382.259 |
|  | 3 | TITLE-ABS-KEY ( "Coronavirus" ) | 273.898 |
|  | 4 | #1 OR #2 OR #3 | 440.025 |
|  | 5 | TITLE-ABS-KEY ( "COVID-19 Vaccines" ) | 20.547 |
|  | 6 | TITLE-ABS-KEY ( "Coronavirus vaccines" ) | 526 |
|  | 7 | #5 OR #6 | 20.845 |
|  | 8 | #4 AND #7 | 20.845 |
|  | 9 | #8 AND PUBYEAR > 2019 AND PUBYEAR < 2023 AND ( LIMIT-TO ( LANGUAGE , "English" ) OR LIMIT-TO ( LANGUAGE , "Spanish" ) ) AND ( LIMIT-TO ( PUBSTAGE , "final" ) ) | 19.350 |
| Identified references | 19.350 | | |

| **Characteristic** | **Report** | | |
| --- | --- | --- | --- |
| Type of Search | New | | |
| Database | Cochrane | | |
| Platform | Cochrane Library | | |
| Date | 23/10/2022 | | |
| Date range search | 2020 - 2022 | | |
| Language restrictions | English-Spanish | | |
| Other limits | Humans | | |
| Search strategy | **Nº** | **Terms** | **Results** |
|  | 1 | MeSH descriptor: [SARS-CoV-2] explode all trees | 1.127 |
|  | 2 | MeSH descriptor: [COVID-19] explode all trees | 2.317 |
|  | 3 | MeSH descriptor: [Coronavirus] explode all trees | 1.144 |
|  | 4 | #1 OR #2 OR #3 | 2.339 |
|  | 5 | MeSH descriptor: [COVID-19 Vaccines] explode all trees | 205 |
|  | 6 | (coronavirus vaccines):ti,ab,kw | 411 |
|  | 7 | #5 OR #6 | 472 |
|  | 8 | #4 AND #7 | 236 |
|  | 9 | #8 AND Filters: Cochrane reviews | 6 |
| Identified references | 6 | | |

**Preferred Reporting Items for Systematic reviews and Meta-Analyses extension for Scoping Reviews (PRISMA-ScR) Checklist**

| **SECTION** | **ITEM** | **PRISMA-ScR CHECKLIST ITEM** | **REPORTED ON PAGE #** |
| --- | --- | --- | --- |
| **TITLE** | | | |
| Title | 1 | Identify the report as a scoping review. | 1 |
| **ABSTRACT** | | | |
| Structured summary | 2 | Provide a structured summary that includes (as applicable): background, objectives, eligibility criteria, sources of evidence, charting methods, results, and conclusions that relate to the review questions and objectives. | 1 |
| **INTRODUCTION** | | | |
| Rationale | 3 | Describe the rationale for the review in the context of what is already known. Explain why the review questions/objectives lend themselves to a scoping review approach. | 2 |
| Objectives | 4 | Provide an explicit statement of the questions and objectives being addressed with reference to their key elements (e.g., population or participants, concepts, and context) or other relevant key elements used to conceptualize the review questions and/or objectives. | 2 |
| **METHODS** | | | |
| Protocol and registration | 5 | Indicate whether a review protocol exists; state if and where it can be accessed (e.g., a Web address); and if available, provide registration information, including the registration number. | 3 |
| Eligibility criteria | 6 | Specify characteristics of the sources of evidence used as eligibility criteria (e.g., years considered, language, and publication status), and provide a rationale. | 3 |
| Information sources* | 7 | Describe all information sources in the search (e.g., databases with dates of coverage and contact with authors to identify additional sources), as well as the date the most recent search was executed. | 4 |
| Search | 8 | Present the full electronic search strategy for at least 1 database, including any limits used, such that it could be repeated. | 4 |
| Selection of sources of evidence† | 9 | State the process for selecting sources of evidence (i.e., screening and eligibility) included in the scoping review. | 4 |
| Data charting process‡ | 10 | Describe the methods of charting data from the included sources of evidence (e.g., calibrated forms or forms that have been tested by the team before their use, and whether data charting was done independently or in duplicate) and any processes for obtaining and confirming data from investigators. | 4 |
| Data items | 11 | List and define all variables for which data were sought and any assumptions and simplifications made. | 4 |
| Critical appraisal of individual sources of evidence§ | 12 | If done, provide a rationale for conducting a critical appraisal of included sources of evidence; describe the methods used and how this information was used in any data synthesis (if appropriate). | NA |
| Synthesis of results | 13 | Describe the methods of handling and summarizing the data that were charted. | 4 |
| **RESULTS** | | | |
| Selection of sources of evidence | 14 | Give numbers of sources of evidence screened, assessed for eligibility, and included in the review, with reasons for exclusions at each stage, ideally using a flow diagram. | 4 |
| Characteristics of sources of evidence | 15 | For each source of evidence, present characteristics for which data were charted and provide the citations. | 5 |
| Critical appraisal within sources of evidence | 16 | If done, present data on critical appraisal of included sources of evidence (see item 12). | NA |
| Results of individual sources of evidence | 17 | For each included source of evidence, present the relevant data that were charted that relate to the review questions and objectives. | Supplementary material |
| Synthesis of results | 18 | Summarize and/or present the charting results as they relate to the review questions and objectives. | 4 - 9 |
| **DISCUSSION** | | | |
| Summary of evidence | 19 | Summarize the main results (including an overview of concepts, themes, and types of evidence available), link to the review questions and objectives, and consider the relevance to key groups. | 9 |
| Limitations | 20 | Discuss the limitations of the scoping review process. | 11 |
| Conclusions | 21 | Provide a general interpretation of the results with respect to the review questions and objectives, as well as potential implications and/or next steps. | 11 |
| **FUNDING** | | | |
| Funding | 22 | Describe sources of funding for the included sources of evidence, as well as sources of funding for the scoping review. Describe the role of the funders of the scoping review. | 12 |

Source: Modified from (1)

Description studies CoronaVac

| **Author and year** | **Intervention (vaccine evaluated)** | **Type of study** | **Country(ies) where it takes place** | **Study population** | **Follow-up time (months)** | **Efficacy/effectivity outcomes** | **Safety outcomes** | **Result** |
| --- | --- | --- | --- | --- | --- | --- | --- | --- |
| Paixao *et al., 2022*(2) | Sinovac (CoronaVac) | Cases and controls | Brazil | Pregnant women | 7 | Prevention of admission to the ICU and/or mechanical ventilation | Not evaluated | 37.4% tested positive for COVID-19, and 7.9% had severe disease. Only 83% of the pregnant women who received the first dose of CoronaVac completed the vaccination scheme. A single dose of the vaccine was not effective in preventing symptomatic COVID-19. The effectiveness of two doses of CoronaVac was 41% (95% CI 27.1–52.2) against symptomatic COVID-19 and 85% (95% CI 59.5–94.8) against severe COVID-19. |
| Deng *et al.,* 2022 (3) | Sinovac (CoronaVac) | Systematic literature review | China | Adults | Not reported | Prevention of infection, of symptomatic COVID-19 and hospital admissions | Not evaluated | The vaccine effectiveness (VE) of the heterologous booster for the prevention of SARS-CoV-2 infection (VE heterologous= 96.10%, VE homologous = 84.00%), of symptomatic COVID-19 (VE heterologous = 56 .80%, VE homologous = 17.30%) and hospital admissions related to COVID-19 (VE heterologous = 97.40%, VE homologous = 93.20%) was higher than the homologous booster vaccine. Compared with the homologous booster group, there was a higher risk of fever (OR = 1.930, 95% CI, 1.199–3.107), myalgia (OR = 1.825, 95% CI, 1.079–3.089), and malaise or fatigue (OR = 1.745, 95% CI, 1.047–2.906) within seven days after booster dose, and higher risk of general malaise or fatigue (OR = 4.140, 95% CI, 1.729–9.916) within 28 days post-booster dose in the heterologous booster group.Compared with the homologous booster group, the geometric mean neutralizing titers (GMTs) of neutralizing antibodies for different SARS-CoV-2 variants and the response rate of antibody and interferon-gamma were higher in the heterologous booster group. |
| Chen *et al.,* 2022 (4) | Sinovac (CoronaVac) | Cohort study | China | Adults | 1, 3, 6 and 12 months | Antibody levels | Not evaluated | All four variants showed similar trends with much lower GMTs (p < 0.0001). The maximum GMTs of the four variants were reached on day 21, 52.5, 78.4, 46.0, and 24.0, respectively. Three months after the booster injection, GMTs decreased significantly to 20.4, 37.5, 22.9, and 6.6; the seropositivity rates decreased to 80.6%, 92.9%, 83.9%, and 42.6% for the Beta, Gamma, Delta, and Omicron variants, respectively. Among the four variants, the GMT of the Gamma variant was the highest (p < 0.0001), and the GMT of the Omicron variant was the lowest (p < 0.0001). No significant differences in GMTs were observed between the Beta and Delta variants (p > 0.9999). |
| Liu *et al.,* 2022 (5) | Sinovac (CoronaVac) | Parallel clinical trial | China | Adults | 1 | Antibody levels | Adverse events post-vaccination | For both vaccines, pain at the injection site is the most reported local AE (incidence rate, IR: 17% in AWcorna vs.. 2% in CoronaVac; P < 0.0001), mostly at Grade 1 level. Fever was the most common systemic AE (IR: 33.5%), followed by headache (IR: 26.0%) and muscle pain (IR: 7.5%) in the AWcorna group. A total of 8 subjects reported Grade 3 fever (IR: 4%) among the 200 participants in the AWcorna group. For the CoronaVac group, headache represented the most frequent systemic AE (IR: 7.0%), followed by fever (IR: 4.0%). No serious adverse events (SAEs) were reported in either group.  83.75% of participants in the AWcorna booster group achieved the 1:8 threshold of neutralizing antibody titers against Omicron compared to only 35% of participants in the CoronaVac booster group (95% Confidence Interval, CI: 30.82 –63.84; P < 0.0001). In addition, RBD-specific IgG antibodies titers also showed a strong increase in both booster groups, and GMTs in the AWcorna booster group were 6.8 and 7.1 fold higher than those in the CoronaVac booster group at both 14-day and 28-day time points respectively (all P < 0.0001). Taken together, these results demonstrate that the heterologous booster with AWcorna induces greater neutralization and IgG antibodies against the WT, Delta, and Omicron variants than the homologous booster |
| Oskay *et al.,* 2022(6) | Sinovac (CoronaVac) | Case report | Turkey | Adults older than 50 years | Not reported | Not reported | Adverse events post-vaccination | Leukocytoclastic vasculitis after the third dose. |
| Hamidreza *et al.,* 2022(7) | Sinovac (CoronaVac) | Systematic literature review | Multicenter | Adults and adolescents | Not reported | Not evaluated | Local adverse events after immunization | RRs of ARs: for inactivated, mRNA, and viral vector vaccines were 1.46 (95% CI: 1.19–1.78), 2.01 (95% CI: 1.82 – 2.23), and 1.65 (95% CI: 1.31 – 2.32). The RR for systemic ARs was 1.13 (95% CI: 0.79 – 1.61), 1.53 (95% CI 1.08 – 2.16), 1.58 (95% CI: 1.13 – 1.90), 0.72 (95% CI: 0.34 – 1.55) and 1.62 (95% CI: 1.39 – 1.89) for inactivated vaccine, mRNA, vector vaccine, DNA vaccine and protein subunits vaccines respectively. The pooled RR of local adverse events after immunization with inactivated vaccine, mRNA vaccine, viral vector vaccine, DNA vaccine, and subunit protein vaccine was 2.18 (95% CI: 1.32 – 3.59), 4.96 (95% CI: 4.02 – 6.11), 1.48 (95% CI: 0.88–2.50), 1.04 (95% CI: 0.12–8.75) and 4.09 (95% CI: 2.63–6.35) respectively. |
| Tong *et al.,* 2022 (8) | Sinovac (CoronaVac) | Cross-sectional | China | Gastrointestinal cancer patients | 8 | Antibody levels | Not evaluated | No serious adverse events (AEs) were found during the follow-up period. Pain at the injection site was the most frequent local non-serious AE (19.75% in the cancer group vs. 20.52% in the healthy group, P = 0.875). Fatigue and headache were the most common systemic AEs. The incidence of these AEs did not differ between the cancer group and healthy controls. After 30 days of observation, no new AEs occurred in either group.  Serum anti-RBD-IgG titers were significantly lower in cancer patients compared with healthy controls (2.18 [IQR: 0.67-5.05] vs. 4.36 [1.31-9.64], P = 0.004). The seroprevalence of anti-RBD-IgG was also lower in cancer patients (70.7% vs. 80.2%, P = 0.047) (Figure 2) in addition to NAbs titers and seroprevalence of NAbs (0.19 [0.13-0.05] vs. 0.31 [0.15-0.54], P = 0.001, 63.7% vs. 75.2%, P = 0.042). |
| Kuloğlu *et al.,* 2022 (9) | Sinovac (CoronaVac) | Cohort study | Turkey | Adults | 5 | Antibody levels | Not evaluated | There was a 3.38-fold increase in neutralizing antibody geometric mean titers (NA GMT, 78.69) 1 month after the Pfizer (BNT) booster and was maintained at three months (NA GMT, 80). However, in the Sinovac-Cornovac (CV) booster group, significantly lower NA GMT than BNT were observed after one month and three months (21.44 and 28.44, respectively) (p < 0.001). In the ELISpot assay, IL-2 levels after BNT were higher than baseline and CV booster (p<0.001), and IFN-γ levels were significantly higher than baseline (p<0.001). 0.001). The CD8+CD38+CD69+ and CD4+CD38+CD69+ T cells were predominantly stimulated in the third month of the BNT booster. |
| Cristelli *et al.,* 2022 (10) | Sinovac (CoronaVac) | Cohort study | Brazil | Kidney transplant recipient patients | 5 | Antibody levels | Not evaluated | COVID-19 incidence rates were similar between seropositive versus seronegative patients (8.6 vs. 7.8 cases/1000 patient-days, P = 0.514); however, the case fatality rate of COVID-19 was significantly higher in those patients with no humoral response after vaccination (18.2% vs. 3.3% in those seropositive, P = 0.009). There were no serious adverse events or acute rejection episodes after vaccination. Before the fourth dose, 46.2% had a negative IgG-anti-SARS-CoV-2 serology. 18.9% of them seroconverted, with a median titer of 78 (IQR 61-189) AU/mL after the booster, with a final seroprevalence of 62.6%. The seroconversion rate among patients who received the fourth dose up to 8 weeks was 22.2%, similar to that observed in those vaccinated >8 weeks after the third dose (17.2%, P = 0.332) |
| Kouhpayeh *et al.,* 2020 (11) | Sinovac (CoronaVac) | Systematic literature review | Unspecified | Adults | Not reported | Reduce rates of infections, seriousness, hospitalization, and mortality | Not evaluated | COVID-19 vaccines have successfully reduced rates of infections, seriousness, hospitalization, and mortality among different populations. The full dose regimen of the Pfizer/BioNTech vaccine is the most effective against B.1.1.7 infections and B.1.351 variants. Despite the high effectiveness of some of the COVID-19 vaccines, further efforts are required to test their effectiveness against other emerging variants |
| Tsun La *et al.,* 2020 (12) | Sinovac (CoronaVac) | Cohort study | China | Adults and adolescents | 1 | Not evaluated | Adverse events post-vaccination | Adverse reactions were reported by 82.7% of those who received Comirnaty and 48.1% of those who received CoronaVac. Compared with Comirnaty, CoronaVac was associated with 83% reduced odds of any adverse reaction [adjusted odds ratio [AOR] = 0.17; 95% confidence interval [CI]: 0.15–0.20], 92% reduced odds of local adverse reactions (AOR = 0.08; 95% CI: 0.06–0.09), and 76% reduced odds of systemic adverse reactions (AOR =0. 24, 95% CI: 0.16–0.28). No significant modification of the effect was identified. |
| Qaderi *et al.,* 2022 (13) | Sinovac (CoronaVac) | Systematic literature review | Saudi Arabia, India, Türkiye and United Kingdom, Italy and Germany | Adults | Not reported | Not evaluated | Injection site adverse reactions and delayed large local reactions | The most common injection site adverse reactions and delayed large local reactions, arising from all vaccine types, were redness/erythema (39%), followed by: itchiness (28%); urticaria rash (17%) on the neck, upper limbs, and trunk; morbilliform eruptions (6.5%); pityriasis rosea (3%); swelling and burning, etc. Most cutaneous reactions occurred in women (84%) and middle-aged people, after the first dose of the vaccine, with onset ranging from 1 to 21 days after vaccination. It appears that cutaneous side effects are more common with mRNA-based COVID-19 vaccines. |
| Kahraman *et al.,* 2022 (14) | Sinovac (CoronaVac) | Cross-sectional | Turkey | Adults | 3 | Not evaluated | Adverse events post-vaccination | 91.6% received CoronaVac and 8% BioNTech. CoronaVac: Systemic reactions at a rate of 31.0% (first dose) and 31.1% (second dose). BioNTech: Systemic reactions at a rate of 46.4% (first dose) and 46.2% (second dose). Malaise at the puncture site: CoronaVac (first dose) 35.6% and (second dose) 35.7%, BioNTech: (first dose) 86.9% and (second dose) 94.1%. One hundred thirty-three non-local cutaneous reactions were observed after the CoronaVac vaccine 2.9% (first dose) and 3.5% (second dose), with the most common being urticaria/angioedema, pityriasis rosea, herpes zoster, and maculopapular rash. After BioNTech, 39 non-local cutaneous reactions were observed 24.8% (first dose) and 5% (second dose); the most common, in order of frequency, were herpes zoster, delayed large local reaction, pityriasis rosea, and urticaria/angioedema. Existing autoimmune diseases were triggered in 2.1% of patients vaccinated with CoronaVac and 8.2% of those vaccinated with BioNTech. |
| Leung *et al.,* 2022 (15) | Sinovac (CoronaVac) | Cross-sectional | China | Children and adolescents with inborn errors of immunity | 2 | Not evaluated | Adverse events post-vaccination | Thirty-nine patients were vaccinated, including 16 with homologous intramuscular 0.3 mL BNT162b2 and 17 with homologous intramuscular 0.5 mL CoronaVac. Two patients received three doses of intradermal 0.5 ml CoronaVac, and 4 patients received two doses of intramuscular BNT162b2 and the third dose of intradermal BNT162b2. No safety concerns were identified.  Inadequate S-RBD IgG and surrogate virus neutralization responses were found after two doses in patients with humoral immunodeficiencies, especially against Omicron BA.1. The third dose of either vaccine increased the S-RBD IgG response. T-cell responses against SARS-CoV-2 antigens were detected in vaccinated IEI patients by intracellular cytokine staining in flow cytometry. The intradermal third-dose vaccine produced a high antibody response in 4 patients. The primary vaccination series of BNT162b2 and CoronaVac in adults and children with IEI should include three doses for optimal immunogenicity. |
| Can *et al.,* 2020 (16) | Sinovac (CoronaVac) | Cohort study | Turkey | Healthcare workers | 3 | Prevention of COVID-19 infection | Not evaluated | 71% of healthcare workers were fully vaccinated, while 29% did not receive any doses. The incidence rate of SARS-CoV-2 infection was 133.7 vs. 70.7 per 100,000 person-days in the unvaccinated and fully vaccinated groups, respectively. The unadjusted effectiveness against COVID-19 infection was 47% (95% CI 31–59%), while the adjusted effectiveness was 39% (95% CI 20–64%) |
| Al-Ali *et al.,* 2022 (17) | Sinovac (CoronaVac) | Systematic literature review | Australia, Austria, Belgium, Canada, China, Denmark, Spain, France, Germany, Greece, India, Ireland, Israel, Italy, Japan, Malaysia, Mexico, Norway, Oman, Poland, Portugal, Qatar, Singapore, Switzerland, United Kingdom, Turkey | Adults | Not reported | Not evaluated | Adverse events post-vaccination | The association between cardiovascular and hematological complications and the five types of vaccines has not been confirmed, and some studies reported that some events may have coincided with the vaccine. There were 1013 events reported in at least 406 individuals who received vaccines from Pfizer, Moderna, and AstraZeneca, as reported by 98 studies. The 1013 events were 14.9% CI, 45.4% thrombosis, 28.3% TP, 10.1% hemorrhage, and 1.3% other cardiovascular and hematological events. Of these, 15.6, 4.4, 73.7, 6.0, and 0.2% were reported after the Pfizer, Moderna, AstraZeneca, J&J, and CoronaVac vaccines, respectively. |
| Ozdede *et al.,* 2022 (18) | Sinovac (CoronaVac) | Cohort study | Turkey | Patients with rheumatic diseases (RD), Behçet syndrome (BS), and familial Mediterranean fever (FMF) | 2 | Not evaluated | Adverse events post-vaccination | BioNTech ensured significantly better efficacy than CoronaVac against COVID-19 in all patient groups (BS: 1.4% vs.. 10.1%; FMF: 3.2% vs. 12.2%, RD: 2.7% vs. 6.4%). People with at least one adverse event (AE) were significantly more frequent among people vaccinated with BioNTech than with CoronaVac (BS: 86.4% vs. 45%; FMF: 83.4% vs. 53.3%; and DR: 83.3% vs. 45.5 %). Most AEs were mild to moderate and transient. Some AEs required medical attention after applying CoronaVac (BS: 5.5%, FMF: 3.3%, and RD: 2.9%) or BioNTech (BS: 5.4 %, FMF: 1.9% and DR: 4.7%) |
| Bostan *et al.,* 2022 (19) | Sinovac (CoronaVac) | Case series | Turkey | Adults | Not reported | Not evaluated | Adverse events post-vaccination | Subacute thyroiditis. |
| Takesaki *et al.,* 2022(20) | Sinovac (CoronaVac) | Cross-sectional | Brazil | Adults older than 50 years | Not reported | Not evaluated | Adverse events post-vaccination | They were able to contact 95.6% and 91.6% of the vaccinated people after the first and second doses, respectively; 31.8% and 23.4% of the contacted participants reported some adverse events (AEs) after the first and second doses, respectively. Pain at the injection site, fatigue, myalgia, and headaches were the most frequent AEs reported. Most of the AEs were mild to moderate. There were eight serious adverse events, but none were considered related to the vaccine. The CoronaVac was safe and well tolerated by these older adults with frailty and comorbidities. |
| Cho *et al.,* 2022 (21) | Sinovac (CoronaVac) | Systematic literature review | South Korea | Patients with solid tumors, hematologic malignant neoplasms, autoimmune diseases, inflammatory bowel disease, and patients that received transplantation or dialysis | Not reported | Antibody levels | Not evaluated | Most solid tumor patients showed a seropositivity rate of more than 80% after the second inoculation. In the type of solid tumor that reported a seropositivity rate of approximately 60% (e.g., esophagus and gastric cancer, neurological cancer), the number of patients was small, so more research is likely needed. Seropositivity of patients with solid tumors was relatively higher than that of patients with hematologic malignant neoplasms or patients with reduced immunity due to transplantation or dialysis. |
| Nantanee *et al.,* 2020 (22) | Sinovac (CoronaVac) | Cohort study | Thailand | Adults | 2 | Not evaluated | Adverse events post-vaccination | The common reactogenicity was erythema at the injection site (53%), while 7% reported blisters. Before the booster, the geometric mean (GM) of sVNT against Delta strain was 22.4% inhibition (95% CI 18.7-26.9), and of anti-S-RBD IgG was 109.3 BAU /ml (95.4-125.1). After the ID booster, GMs of sVNT against Delta strain were 95.5% inhibition (95%CI 94.2–96.8) at day 14, 73.1% inhibition (66.7–80.2) at day 90, and 22.7% inhibition (14.9–34.6) at day 180. Differences in the proportion of participants achieving sVNT against Delta strain ≥ 80% inhibition in ID recipients vs. IM were +4.2% (95% CI -2.0 to 10.5) at day 14 and -37.3% (-54.2 to -20.3) at day 90. Anti-S-RBD IgG GMs were 2037.1 BAU/ml (95%CI 1770.9–2343.2) at day 14 and 744.6 BAU/ml (650.1–852.9) at day 90, respectively. Geometric mean ratios (GMRs) of anti-S-RBD IgG were 0.99 (0.83–1.20) at day 14 and 0.82 (0.66–1.02) at day 90. Only 18% reported fever, compared with 37% of IM (p = 0.003) |
| Kolahchi *et al.,* 2022 (23) | Sinovac (CoronaVac) | Systematic literature review | Iran | Adults | Not reported | Not evaluated | Adverse events post-vaccination | Twenty-nine studies (43 patients) were included: 22 patients (51.1%) were diagnosed with AIS associated with vaccine-induced immune thrombotic thrombocytopenia (VITT). Only one of the AIS associated with the VITT group received viral vector vaccines. Most cases with AIS and VITT were women (17 cases, 77.2%) aged below 60 years (15 cases, 68%). Fourteen patients (32.5%) had additional thrombosis in other sites. Four of them (0.09%) showed concurrent CVST and ischemic stroke. Hemorrhagic transformation following AIS occurred in 7 patients (16.27%). Among 43 patients with AIS, at least six patients (14%) died during hospital admission |
| Clemens *et al.,* 2022 (24) | Sinovac (CoronaVac) | Parallel clinical trial | Brazil | Adults | 6 | Antibody levels | Adverse events post-vaccination | There were five serious adverse events. Three were considered possibly related to the vaccine received: one in the Pfizer group and two in the Janssen group. All participants recovered and were discharged home.  Antibody concentrations were low at six months after immunization with two doses of CoronaVac. However, all four vaccines administered as a third dose significantly increased binding and neutralizing antibodies, which could improve protection against infection. The heterologous booster resulted in more robust immune responses than the homologous booster and could enhance protection. |
| Kang *et al.,* 2022 (25) | Sinovac (CoronaVac) | Cohort study | China | Cancer patients | 8 | Not evaluated | Adverse events post-vaccination | Among patients with active cancer, the incidence of adverse events of special interest - AESI was 0.31 and 1.02 per 10,000 person-days with BNT162b2 vs. unvaccinated patients and 0.13 and 0.88 per 10,000 person-days with CoronaVac vs. unvaccinated patients. Among patients with a history of cancer, the incidence was 0.55 and 0.89 per 10,000 person-days with BNT162b2 vs. unvaccinated patients and 0.42 and 0.93 per 10,000 person-days with CoronaVac vs. unvaccinated patients. Neither vaccine was associated with an increased risk of AESI for patients with active cancer (BNT162b2: HR 0.30, 95%CI 0.08–1.09; CoronaVac: 0.14, 95%CI 0.02–1.18) or with a history of cancer (BNT162b2: 0.62, 95% CI: 0.30–1.28, CoronaVac: 0.45, 95% CI: 0.21–1.00). |
| Lin *et al.,* 2022 (26) | Sinovac (CoronaVac) | Systematic literature review | China | Adults | Not reported | Antibody levels | Adverse events post-vaccination | Fatigue, headache, and pain at the injection site were the most frequent systemic and local reactions. Assessment of Grade 3 and Grade 4 adverse events in participants that previously received Pfizer-BNT162b2/BNT162b2 or Astrazeneca-AZD1222/AZD1222 as a subgroup reveals that a different booster vaccination after two doses of BNT162b2 was associated with a higher risk of Grade 3 and Grade 4 adverse events (risk ratio [RR], 1.97; 95% confidence interval [CI], 1.17–3.33), while no statistical difference was found in a different booster vaccination after two doses of AZD1222 compared with control (RR, 1.34; 95% CI, 0.84–2.15) (Figure 1). In all of these studies, no deaths associated with the COVID‐19 booster vaccines were documented. All booster vaccinations showed acceptable side-effect profiles in all three studies, although some schedules showed more than others.  These studies reported that people vaccinated with 1) inactivated SARS‐CoV‐2 virus VLA2001 (Valneva; hereafter referred to as VLA), mRNA vaccine mRNA1273 (Moderna; hereafter referred to as m1273), mRNA vaccine CVnCov (CureVac; hereafter referred to as CVn), viral vector vaccine ChAdOx1 nCov‐19 (ChAd; AZD1222; Oxford–AstraZeneca; hereafter referred to as AZD1222), nanoparticle vaccine NVX‐CoV2373 (Novavax; hereafter referred to as NVX), or replication‐deficient adenovirus vector vaccine Ad26.COV2.S (Janssen; hereafter referred to as Ad26) after BNT162b2/BNT162b2; 2) BNT162b2, m1273, CVn, NVX or Ad26 after AZD1222/AZD1222; 3) inactivated vaccine BBIBP‐CorV (Sinopharm; hereafter referred to as BBIBP), BNT162b2 and AZD1222 after CoronaVac/CoronaVac could significantly boost antibody responses on day 28 after the third dose. |
| Shinjo, et al., 2021 (27) | Sinovac (CoronaVac) | Cases and controls | Brazil | Patients with systemic autoimmune myopathies (SAM) | 3 | Antibody levels | Adverse events post-vaccination | Patients and CTRL were of comparable sex (P>0.99) and age (P=0.90). Vaccine-related adverse events were mild, with similar frequencies in patients and controls (P>0.05).  Immunogenicity of 37 patients and 79 CTRL naïve participants revealed at D69 a moderate but significantly lower seroconversion (64.9% vs. 91.1%, P<0.001), anti-S1/S2 IgG geometric mean titer (GMT) [7.9 (95%CI 4.7-13.2) vs. 24.7 (95%CI 30.0-30.5) AU/ml, P<0.001] and frequency of neutralizing antibody (NAb) positivity (51.4% vs. 77.2%, P<0.001) in cases compared to controls. Type of SAMs, disease status, other drugs, or comorbidities did not influence immunogenicity. |
| Bostan *et al.,* 2022 (28) | Sinovac (CoronaVac) | Cross-sectional | Turkey | Adults | 10 | Not evaluated | Adverse events post-vaccination | Of the study population, 16 (29.1%) were diagnosed with COVID-19 vaccine-associated subacute thyroiditis (SAT) (10 with CoronaVac® and 6 with the Pfizer-BioNTech® vaccine), with a median time from onset of symptoms after vaccination of 6.5 (range, 2–20) days. There were no statistically significant differences between the vaccine-associated (VA) and non-vaccine-associated (NVA) groups in terms of age, sex, time to diagnosis, thyroid volumes, thyroid function tests, and acute phase reactant. |
| Cao et al., 2022 (29) | Sinovac (CoronaVac) | Cohort study | China | Patients with allergic diseases | 1 | Antibody levels | Adverse events post-vaccination | Pain at the injection site was the most reported local reaction, happening in 31.0% of patients with allergic disease and 18.9% in the control group, respectively (P < 0.001). After the first dose, systemic events were reported more frequently in patients with allergic disease than in the control group (30.2% vs. 22.9%, P < 0.001). After the second dose, systemic events occurred less frequently, affecting 17.1% of the patients and 11.1% of the control group (P < 0.002). The appearance of fatigue, vertigo, diarrhea, skin rash, and sore throat were the most frequent systemic reactions.  Almost all participants were IgG antibody-positive, and participants with allergic diseases had higher frequencies than those in the control group (100.0 vs. 99.4% |
| Dundar et al., 2022 (30) | Sinovac (CoronaVac) | Cohort study | Turkey | Healthcare workers | 2 | Prevention of COVID-19 infection and Antibody levels | Not evaluated | The seropositivity rate post-vaccine was 97.5% (n = 117) of all subjects, and 44 were seropositive after the first dose. The percentage of prior COVID-19 infection (59.1%) among seropositive individuals before the second vaccination was significantly higher than that of seropositive individuals (10.96%) after the second vaccination (p < 0.0001).  Anti-SARS-CoV-2 antibody responses in people infected with SARS-CoV-2 follow a classic pattern, with a rapid increase within the first three weeks after symptom onset. |
| Jara et al., 2022 (31) | Sinovac (CoronaVac) | Cohort study | Chile | Adults | 9 | Prevention of infection, of symptomatic COVID-19 and hospital admissions | Not evaluated | Vaccine effectiveness (VE) against symptomatic COVID-19 with CoronaVac was 78.8% (95% CI 76.8-80.6); with BNT162b2 was 96.5% (96.2-96.7) and with AZD1222 was 93.2% (92.9-93.6). The VE of CoronaVac against hospitalization was 86.3% (83.7-88.5), admission to the ICU 92.2% (88.7-94.6), and against COVID-19-associated death 86.7% (80, 5-91.0). |
| Netto et al., 2022 (32) | Sinovac (CoronaVac) | Cohort study | Brazil | Immunosuppressed patients | 3 | Antibody levels | Not evaluated | IgG titers were 48.7 AU/mL (IQR 26.6-88.2) in people with HIV compared with 75.2 AU/mL (IQR 50.3-112.0) in people without previously known immunosuppression (p<0 0001). |
| Cerqueira-Silva *et al.,* 2022 (33) | Sinovac (CoronaVac) | Cohort study | Brazil | Adults | 4 | Prevention of hospital admissions | Not evaluated | Vaccine effectiveness (VE) was 84.1% (95%CI: 83.2-84.9), with VE decreasing from 81.3% (95%CI: 77.9-84.2) at 31-60 days to 72,9% (95% CI: 70.6-75.1) at 120 days or more after the booster dose. |
| McMenamin *et al.,* 2022 (4) | Sinovac (CoronaVac) | Cohort study | China | Adults | 4 | Prevention of death | Not evaluated | Vaccine effectiveness of BNT162b2 in adults 60 years or older was 89.3% (95%CI=86.6-91.6) compared to 69.9% of CoronaVac (95%CI=64.4-74.6). Three doses of either vaccine offered very high levels of protection against severe or fatal outcomes VE=97.9% (95%CI=97.3-98.4). |
| Han et al., 2022 (5) | Sinovac (CoronaVac) | Umbrella clinical trial | China | Cancer patients | 7 | Antibody levels | Adverse events post-vaccination | Ten (10.2%) patients had side effects after SARS-CoV-2 vaccination. Among them, two (2.0%) patients had a fever, five (5.1%) patients had injection site pain, and one (1.0%) patient felt dizzy.  SARS-CoV-2 IgG antibodies’ positivity of two doses of CoronaVac or BBIBP-CorV vaccine was 63.0% (46/73); 4 months after the last vaccination dose, the IgG-positive rate (31.6%, 6/19) decreased significantly in patients with thyroid cancer. The IgG-positive rate (81.0%, 64/79) was satisfactory within three months after the last vaccination dose. |
| Ai *et al.* 2022 (6) | Sinovac (CoronaVac) | Umbrella clinical trial | China | Patients with chronic liver disease | 8 | Antibody levels | Adverse events post-vaccination | Most adverse reactions were mild and transient, with pain at the injection site (n = 36; 8.2%) being the most frequently reported adverse event. Three participants had a Grade 3 aminotransferase elevation (defined as alanine aminotransferase > 5 upper limits of normal) after the second dose of inactivated whole-virion SARS-CoV-2 vaccine, and only 1 of them was considered a serious adverse event potentially related to SARS-CoV-2 vaccination.  Positive rates for SARS-CoV-2 neutralizing antibodies were 76.8% in the non-cirrhotic CLD group, 78.9% in the compensated cirrhotic group, 76.7% in the decompensated cirrhotic group (p = 0.894 among CLD subgroups) and 90.3% in healthy controls. (p = 0.008 vs. CLD group). |
| Fu et al., 2022 (7) | Sinovac (CoronaVac) | Systematic literature review | China | Children | Not reported | Determine the efficacy, effectiveness, and cost-effectiveness | Not evaluated | A total of 24 studies were included in the meta-analysis. Compared with no immunization, the effectiveness of an inactivated vaccine against COVID-19 infection, hospitalization, ICU admission, and death were 65.18% (95% CI 62.62, 67.75), 79.10% (95% CI 71.69, 86.51), 90.46% (95% CI 89.42, 91.50), and 86.69% (95% CI 85.68, 87.70); and efficacy against COVID-19 infection and hospitalization were 70.56% (95% CI 57.87, 83.24) and 100% (95% CI 61.72, 100). Vaccination with inactivated vaccines prevented more infections, hospitalizations, ICU admissions, and deaths with lower total costs; therefore, it was a cost-saving from a societal perspective in China. |
| Ciampi et al., 2022 (8) | Sinovac (CoronaVac) | Umbrella clinical trial | Chile | Multiple sclerosis patients | 6 | Antibody levels | Adverse events post-vaccination | The humoral response was observed in 66.9% (Inactivated vaccine 62.6% vs. 78.4% mRNA vaccine, p = 0.04). Positive anti-S1 antibodies were observed in 100% of patients with no DMT (n=3), 100% with interferon/glatiramer-acetate (n=11), 100% with teriflunomide/dimethyl-fumarate (n=16), 100% with natalizumab (n = 10), 100% with alemtuzumab (n = 8), 90% with cladribine (n = 10) and 88% with fingolimod (n = 17), while 43% of patients who received anti-CD20 (n=99) were positive (38% inactivated vaccine vs. 59% mRNA vaccine, p=0.05). |
| Yasin et al., 2022 (9) | Sinovac (CoronaVac) | Cohort study | Turkey | Cancer patients and healthy patients | 1 | Prevention of hospital admissions | Adverse events post-vaccination | This study highlighted the efficacy and safety of the inactivated vaccine in cancer patients. The seropositivity rate was 85.2% in the patient group and 97.5% in the control group. The seropositivity rate and antibody levels were significantly lower in the patient group (p < 0.001). Age and chemotherapy were associated with lower seropositivity in cancer patients (p < 0.001). |
| Lu *et al.,* 2022 (10) | Sinovac (CoronaVac) | Umbrella clinical trial | China | Tuberous sclerosis complex patients with epilepsy | 7 | Not evaluated | Adverse events post-vaccination | To assess the safety of the inactivated vaccine in tuberous sclerosis complex (TSC) patients with epilepsy: Adverse reactions occurred within 28 days after injection in 11 patients (25%), all under 12 years of age. Pain at the injection site was the most reported event (20.45%), which was mild in severity and improved within one day. All patients had no seizure-related changes after vaccination. |
| Cerqueira-Silva et al., 2022 (11) | Sinovac (CoronaVac) | Cases and controls | Brazil | Adults | 9 | Prevention of infection, hospital admissions and death | Not evaluated | Vaccine effectiveness against symptomatic infection 14 or more days after completing the vaccination schedule was 39.4% (95% CI 36.1-42.6) for CoronaVac, 56.0% (51.4-60.2) for ChAdOx1 nCoV-19, 44.0% (31.5-54.2) for Ad26.COV2.S, and 64.8% (54.9-72.4) for BNT162b2. For the two-dose vaccine schedule (CoronaVac, ChAdOx1 nCoV-19, and BNT162b2), efficacy against symptomatic infection was significantly higher after the second dose than after the first. The effectiveness against hospitalization or death 14 or more days after the completion of the vaccination schedule was 81.3% (75.3-85.8) for CoronaVac, 89.9% (83.5-93.8) for ChAdOx1 nCoV-19, 57.7% (-2.6 to 82.5) for Ad26.COV2.S, and 89.7% (54.3-97.7) for BNT162b2. |
| Karamese and Tutuncu, 2022 (12) | CoronaVac | Cross-sectional | Turkey | Adults older than 50 years | 2 months | “57.02% had an antibody level after four weeks from the first dose of the vaccine”. | “Vaccine showed good safety” | Mean levels of anti-SARS-CoV-2 IgG antibodies four weeks after the first and second vaccine doses were 37.70 ± 57.08 IU/ml and 194.61 ± 174.88 IU/ml, respectively. Furthermore, 134 of 235 participants (57.02%) had an antibody level of less than 25.6 IU/ml (negative) after four weeks from the first dose of the vaccine, while this rate was 11.48% (n = 27) four weeks after the second dose of the vaccine. The 19 (70.4%) participants who had an antibody level less than 25.6 IU/mL four weeks after the first dose of the vaccine had at least one comorbid disease, including diabetes mellitus, and 8 (29 .6%) participants did not have any comorbid disease (F=2.352, p=0.006). |
| Bueno et al., 2022 (13) | CoronaVac | Crossover clinical trial | Chile | Adults | 2 and 4 weeks | “Seroconversion rates for specific anti-S1-receptor binding domain (RBD) immunoglobulin G (IgG) were 82.22% and 84.44% in the 18–59 year age group and 62.69% and 70.37% in the ≥60 year age group, after the second dose, respectively. A significant increase in circulating neutralizing antibodies was detected 2 and 4 weeks after the second dose”. | “The most reported solicited local advers events was pain at the injection site (mostly grade 1) (55.6% in the vaccine arm compared with 40.0% in the placebo arm). Headaches (grade 1 or 2) with a frequency of 48.5% in the vaccine arm and 48.8% in the placebo arm.” | The main adverse reaction in the 434 volunteers was pain at the injection site, with a higher incidence in the vaccine arm than in the placebo arm. The adverse reactions observed were mostly mild and local. No serious adverse events were reported.  Seroconversion rates for specific anti-S1-receptor binding domain (RBD) immunoglobulin G (IgG) were 82.22% and 84.44% in the 18 to 59 years age group and 62.69% and 70.37 % in the ≥60-year age group. |
| Demirbakan *et al.,* 2022 (14) | CoronaVac | Cohort study | Turkey | Healthcare workers | 3 months | “Anti-spike antibodies in 834/1072 (77.8%) healthcare workers. Seropositivity was higher among women (84.6%) than among men (70.6% p < 0.001)” | Not evaluated. | Four weeks after the first dose of the vaccine, anti-spike antibodies were detected in 834/1072 (77.8%) of the HCWs. Seropositivity was higher among women (84.6%) than among men (70.6% p < 0.001), and it was found to be higher in both women and men between the ages of 18 to 34 years. Anti-spike antibodies were detected in 1008 of 1012 (99.6%) 21 days after the second dose and 803 of 836 (96.1%) 3 months after the second dose. Immunogenicity decreased significantly (p < 0.001) 3 months after the second dose of the vaccine. |
| Dong *et al.,* 2022 (15) | CoronaVac | Cohort study | China | Adults | 4 months | “No significant differences were found in other laboratory results and pregnancy outcomes between the four groups (p > 0.05)”. | Not evaluated, however is mentione, “One reason for the vaccine safety concerns is that there may be homology between the placental syncytin-1 protein and the COVID-19 spike protein targeted by the vaccine, which could lead to infertility” | No significant differences were found in other laboratory results and pregnancy outcomes between the four groups (p > 0.05). |
| Kara et al., 2022 (16) | CoronaVac | Parallel clinical trial | Turkey | Adults | 2 months | “In patients with severe obesity, with and without prior SARS-CoV-2 infection, spike antigen antibody levels of those vaccinated with BNT162b2 were found to be signifcantly higher than those vaccinated with CoronaVac (p=0.043, p<0.001 respectively)”. | Not evaluated | Antibody titers against SARS-CoV-2 spike antigen of severely obese patients were significantly lower than normal weight controls (p = 0.001, p = 0.001, respectively). In seventy subjects with prior SARS-CoV-2 infection, spike antigen antibody titers in severely obese patients vaccinated with BNT162b2 or CoronaVac were not significantly different from normal weight controls (p = 0.1, p = 0.1 respectively). |
| Bilgin et al., 2022 (17) | CoronaVac | Parallel clinical trial | Turkey | Healthcare worker | 3 months | “The Anti-S antibodies were detectable in all healthcare workers (n: 224). The median Anti-S titers (BAU/mL) was significantly higher in SARS-CoV-2 infection (VAI) (620 25–75% 373–1341) compared to SARS-CoV-2 infection only (VO) (136, 25–75% 85–283) and , SARS-CoV-2 infection only (IO) (111, 25–75% 54–413, p < 0.01). VAI group had significantly lower percentage of plasmablasts (2.9; 0– 8.7) compared to VO (6.8; 3.5–12.0) and IO (9.9; 4.7–47.5, p < 0.01) (n:74). Percentage of long-lived plasma cells (LLPC) in groups VO, VAI and IO was similar”. | Not evaluated | The median of Anti-S titers (BAU/mL) was significantly higher with the vaccination-after-infection group (620 25-75% 373-1341) compared to the vaccine-only group (136, 25-75% 85-283) and SARS-CoV-2 infection only group (111, 25-75% 54-413, p < 0.01). The vaccination-after-infection group had a significantly lower percentage of plasmablasts (2.9; 0-8.7) compared to vaccine-only (6.8; 3.5-12.0) and infection only (9.9; 4.7-47.5, p < 0.01) (n: 74) groups. |
| Caironi *et al.,* 2022 (18) | CoronaVac | Systematic literature review | United States, Turkey, Spain, Italy, Greece, Japan, South Korea, and the United Kingdom | Adults | 1 month | Does not apply | “The thyroid diseases described in the analyzed literature were 59 subacute thyroiditis (SAT), 29 Graves’ disease (GD) (4 of which were relapses or worsening of pre-existent GD), 2 co-occurrence of SAT and GD, 6 painless thyroiditis (PT), one of which associated with thyrotoxic periodic paralysis, 1 thyroid eye disease (TED), 1 hypothyroidism associated with mixedema, and 1 case described as generic thyroiditis”. | The thyroid disorders described in the reviewed literature were 59 subacute thyroiditis (SAT); 29 Graves' disease (GD) (4 of which were relapses or worsening of pre-existing GD); 2 co-occurrence of SAT and GD; 6 painless thyroiditis (PT), one of which associated with thyrotoxic periodic paralysis; one thyroid eye disease (TED); one hypothyroidism associated with myxedema, and one case described as generic thyroiditis. |
| Cheung *et al.,* 2022 (19) | CoronaVac and Pfizer | Cohort study | Hong Kong, China | Adults | 6 months | “For CoronaVac (n=67, 22.7%), there was no statistical differences  in seroconversion rates (day21: 7.1% vs. 15.1%; day56: 64.3% vs. 83.0%) or vMN GMT (5.3 vs. 5.8,) at day28. However,  moderate/severe HS patients had lower vMN GMT (9.1 vs. 14.8, P=0.021) at day 56 with lower proportion having highesttier response (21.4% vs. 52.8%, P=0.036)”. | “One hundred ninety (83.3%) Pfizer recipients and 36 (53.7%) CoronaVac recipients reported adverse events within 7 days of first dose of vaccine (Supplementary Table 2). All adverse events were mild (grade 1 and 2) and self-limiting. The most common local and systemic adverse events were injection site pain (78.1% for BNT162b2 and 35.8% for CoronaVac) and fatigue (30.7% BNT162b2 and 28.4% for CoronaVac)”. | For Pfizer (n=228, 77.3%), there was no statistical difference in seroconversion rates (day 21: 71.7% vs. 76.6%; day 56: 100% vs. 100%) or microneutralization (vMN) geometric mean titer (GMT) (day 21: 13.2 vs. 13.3, day 56: 91.9 vs. 101.4) between moderate/severe hepatic steatosis (HS) and control groups, respectively. A smaller proportion of patients with moderate/severe HS had a highest-tier response (Day 56: 5.0% vs. 15.5%; P = 0.037). For CoronaVac (n=67, 22.7%), there were no statistical differences in seroconversion rates (day 21: 7.1% vs. 15.1%; day 56: 64.3% vs. 83.0%) or vMN GMT (5.3 vs. 5.8) at day 28. |
| Cohen et al., 2022 (20) | CoronaVac | Cohort study | Thailand | Adults | 1 month | “The geometric mean ratio (GMR) of the antireceptor binding domain (RBD) antibody concentration 4 weeks after the booster dose was 2.58 (95% confidence interval [CI], 1. 80–3.71) and 8.69 (95% CI: 6.05–12.47) in the AZD1222/CoronaVac and CoronaVac/AZD1222 cohorts, respectively. Reactogenicity was similar after initial and booster doses with the same vaccine”. | Not evaluated, however, the paper mentions: “All participants were observed for at least 15 minutes after each vaccination to monitor immediate adverse events” | The geometric mean ratio (GMR) of the anti-receptor binding domain (RBD) antibody concentration four weeks after the booster dose was 2.58 (95% confidence interval [CI]: 1. 80–3.71) and 8.69 (95% CI: 6.05–12.47) in the AZD1222/CoronaVac and CoronaVac/AZD1222 cohorts, respectively. The reactogenicity was similar after the initial and booster doses with the same vaccine. |
| Ranzani et al., 2022 (21) | CoronaVac | Cases and controls | Brazil | Adults | 2 months | “Inactivated vaccines (VE) against symptomatic disease was 8.6% (95% CI, 5.6–11.5) and 56.8% (95% CI, 56.3–57.3) in the period 8–59 days after receiving a homologous and heterologous booster, respectively. During the same interval, VE against severe Covid-19 was 73.6% (95% CI, 63.9–80.7) and 86.0% (95% CI, 84.5–87.4) after receiving a homologous and heterologous booster, respectively”. | Not evaluated | Vaccine effectiveness against symptomatic disease was 8.6% (95% CI, 5.6–11.5) and 56.8% (95% CI, 56.3–57.3) in the 8-59 days after receiving a homologous and heterologous booster, respectively. Over the same interval, the vaccine effectiveness against severe COVID-19 was 73.6% (95% CI, 63.9–80.7) and 86.0% (95% CI, 84.5–87.4) after receiving a homologous and heterologous booster. |
| Li *et al.,* 2021 (22) | CoronaVac | Cases and controls | China | Adults | 1 month | “After adjusting for age and sex, the overall vaccine effectiveness (VE) for two-dose vaccination was 59.0% (95% confidence interval: 16.0% to 81.6%) against coronavirus disease 2019 (COVID-19) and 70.2% (95% confidence interval: 29.6–89.3%) against moderate COVID-19 and 100% against severe COVID-19 which might be overestimated due to the small sample size.” | Not evaluated | Vaccination status was compared to estimate the vaccine effectiveness (VE) of SARS-CoV-2 inactivated vaccines. A single dose of inactivated SARS-CoV-2 vaccine produced a VE of only 13.8%. After adjusting for age and sex, the overall VE for the two-dose vaccination was 59.0% (95% confidence interval: 16.0% to 81.6%) against coronavirus disease 2019 (COVID-19) and 70.2% (95% confidence interval: 29.6% to 89.3%) against moderate COVID-19. The VE of the two-dose vaccination against COVID-19 reached 72.5% among participants aged 40 to 59 years. |
| Cheng *et al.,* 2022 (23) | CoronaVac and BNT162b2 | Cohort study | China | Adults | 4 months | “With neutralization of the virus (SARS-COV2), those vaccinated with Pfizer had reductions of 4.8, 3.4, 4.6, 11.3 and 15.5 times in the geometric mean antibody titers (GMT) to BA.1, BA.2, BA.2.12.1, BA. 4 and BA.5 virus, respectively. Similarly, those vaccinated with CoronaVac (Sinovac) had GMT reductions of 8.0, 7.0, 11.8, 12.0, and 12.0-fold and those with two doses of CoronaVac boosted with BNT162b2 had GMT reductions of 6.1, 6.7, 6.3, 13.0 and 21.2 times, respectively” | Not evaluated | Reduced serum antibodies against the Omicron variant (geometric mean titer (GMT) < 10) compared to wild-type (WT) virus 3 to 5 weeks after two doses of BNT162b2 vaccine (GMT = 218.8) or CoronaVac (GMT = 32.5). A booster dose of BNT162b2 elicited Omicron PRNT_50_ titers ≥25.6 in 88% of subjects (22 of 25) who had previously received two doses of BNT162b2 and in 80% of subjects (24 of 30) who had previously received CoronaVac. However, few people who were previously infected (3%) (1 of 30) or those vaccinated with three doses of CoronaVac (1 of 30) reached this threshold. |
| Jara et al., 2022 (24) | CoronaVac | Cohort study | Chile | Children | 3 months | “Estimated efficacy against COVID-19 was 38.2% (95% CI, 36.5–39.9), against hospitalization 64.6% (95% CI, 49.6–75.2), and to prevent intensive care unit admission 69.0% (95% CI, 18.6–88.2).” | Not evaluated | Estimated efficacy against COVID-19 was 38.2% (95% CI, 36.5–39.9), against hospitalization 64.6% (95% CI, 49.6–75.2), and to prevent intensive care unit admission 69.0% (95% CI, 18.6–88.2). |
| Cerqueira-Silva et al., 2022 (25) | CoronaVac and BNT162b2 | Cohort study | Brazil | Adults | 4 months | “Compared to unvaccinated, vaccinees maintained protection against severe outcomes, with an estimated vaccine effectiveness (VE) of 84.1% (95% CI:83.2–84.9) at more than 120 days after BNT162b2 booster.” | Not evaluated | The vaccine effectiveness (VE) of CoronaVac at 30 days after the second dose was 55.0% (95% confidence interval (CI) 54.3–55.7) against confirmed infection, and 82.1 % (95% CI: 81.4–82.8) against severe outcomes. VE decreased to 34.7% (95% CI: 33.1–36.2) against infection and 72.5% (95% CI: 70.9–74.0) against severe outcomes over 180 days after the second dose. A Pfizer booster, six months after the second dose of CoronaVac, improved VE against infections to 92.7% (95% CI: 91.0-94.0) and VE against severe outcomes to 97.3% (95% CI: 96.1-98.1) 30 days after the booster. |
| Simsek et al., 2022 (26) | CoronaVac and BNT162b2 | Cohort study | Turkey | Adults | 12 months | “In in‐group comparisons of full‐dose vaccinated patients, while no to Covid‐19 occurrence, the difference was observed between two doses of BNT162b2 (Pfizer–BioNTech) and three doses of CoronaVac (p= 0.432), a statistically significant difference was observed between all other groups (p< 0.005). When the data of 578 patients who experienced Covid‐19 was analyzed, a statistically significant difference was observed between the groups who were full‐dose vaccinated and those who were not (p= 0.000)” | Not evaluated | 81.2% of patients were fully vaccinated, and 18.8% did not receive the full vaccination dose. A statistically significant difference in the occurrence of COVID-19 was found between patients who did and did not receive the full-dose vaccination (p = 0.000). In comparisons within the group of full‐dose vaccinated patients, no differences were observed between two doses of BNT162b2 (Pfizer–BioNTech) and three doses of CoronaVac (p = 0.432). |
| Fernandes et al., 2021 (27) | CoronaVac | Cohort study | Brazil | Children | 1 month | “Of 27 children tested 3-9 days after vaccination, 5 (19%) had positive serology suggesting a previous natural SARS-CoV-2 infection, with all 19 tested on day 30 after vaccination and presenting with positive tests, with an increment of antibody titers in those initially positive”. | “All monitored  children did not show adverse events following the  immunization” | After the first dose, 18.5% had a total S1 protein IgG titer greater than 1.0 (reagent tests) 3-9 days post-vaccination. One month after vaccination, all had total Spike S1 protein IgG titers higher than 1.0. About half (47%) tested for the receptor-binding domain inhibition (RBI) showed results greater than 20%, but most had low binding inhibition (5-20%), with only three cases with high titers (over 90% inhibition). |
| Ranzani et al., 2021 (28) | CoronaVac | Cases and controls | Brazil | Adults | 1 month | “The adjusted vaccine effectiveness against symptomatic COVID-19 was 24.7% (95%CI, 14.7% to 33.4%) at 0-13 days and 46.8% (38.7% to 53.8%) at ≥14 days after the second dose. The adjusted vaccine effectiveness against hospital admissions was 55.5% (95%CI 46.5% to 62.9%), and against deaths was 61.2% (95%CI 48.9% to 70 .5%) at ≥14 days after the second dose”. | Not evaluated | The adjusted vaccine effectiveness against symptomatic COVID-19 was 24.7% (95%CI, 14.7% to 33.4%) at 0-13 days and 46.8% (38.7% to 53.8%) at ≥14 days after the second dose. The adjusted vaccine effectiveness against hospital admissions was 55.5% (95%CI 46.5% to 62.9%), and against deaths was 61.2% (95%CI 48.9% to 70 .5%) at ≥14 days after the second dose. |
| Duarte et al., 2021 (29) | CoronaVac | Parallel clinical trial | Chile | Adults | 1 month | “Effectiveness of 65.9% for symptomatic cases. Although vaccination reduces the risk of disease, infections can still occur during or after completion of the vaccination schedule (breakthrough cases)” | “Forty-five subjects reported a symptomatic SARS-CoV-2 infection 14 or more days after the second dose (1.99% of fully vaccinated subjects). 96% of them developed a mild disease, one case developed a moderate disease, and one developed a severe disease and required mechanical ventilation”. | Forty-five subjects reported a symptomatic SARS-CoV-2 infection 14 or more days after the second dose (1.99% of fully vaccinated subjects). 96% of them developed a mild disease, one case developed a moderate disease, and one developed a severe disease and required mechanical ventilation. |
| Wu *et al.,* 2021 (30) | CoronaVac | Parallel clinical trial | China | Adults older than 50 years | 1 month | “In phase II, seroconversion was observed in 88 of 97 participants in the 1.5 μg group (90.7% [83.1-95.7]), in 96 of 98 in the 3 μg group (98.0% [92.8-99.8]), and in 97 of 98 (99 0% [94.5-100.0]) in the 6 μg group”. | “Adverse reactions occurred in 20 (20%) of 100. The most common reactions  were injection site pain (39 [9%] participants) and  fever (14 [3%])” | Adverse reactions occurred in 20 (20%) of 100 participants in the 1·5 μg group, 25 (20%) of 125 in the 3 μg group, 27 (22%) of 123 in the 6 μg group, and 15 (21%) of 73 in the placebo group. In phase II, seroconversion was observed in 88 of 97 participants in the 1.5 μg group (90.7% [83.1-95.7]), in 96 of 98 in the 3 μg group (98.0% [92.8-99.8]), and in 97 of 98 (99 0% [94.5-100.0]) in the 6 μg group. |
| Jara et al., 2021 (31) | CoronaVac | Cohort study | Chile | Adults | 3 months | “Adjusted vaccine effectiveness was 65.9% (95%CI, 65.2 to 66.6) for preventing laboratory-confirmed COVID-19, 87.5% (95%CI, 86.7 to 88.2) for preventing hospitalization, 90.3% (95% CI, 89.1 to 91.4) for preventing admission to ICU and 86.3% (95%CI, 84.5 to 87.9) for preventing death.” | Not evaluated | Adjusted vaccine effectiveness was 65.9% (95%CI, 65.2 to 66.6) for preventing laboratory-confirmed COVID-19, 87.5% (95%CI, 86.7 to 88.2) for preventing hospitalization, 90.3% (95% CI, 89.1 to 91.4) for preventing admission to ICU and 86.3% (95%CI, 84.5 to 87.9) for preventing death. |
| Ling *et al.,* 2021 (32) | CoronaVac | Systematic literature review | China | Adults | 1 month | “The time point for testing the effectiveness of the vaccine is relatively short, and it is temporarily impossible to know how long the effect of the vaccine against SARS-CoV-2 can last.” | “Adverse events were significantly higher in the inactivated virus vaccine group than in the placebo group (OR: 2.44, 95%CI: 0.76~7.87, I2 =83%), and local adverse events were higher than those in the placebo group (OR: 1.22, 95%CI: 0.87~1.71, I2 =0%), systemic adverse events were less different from those in placebo group (OR: 0.92, 95%CI: 0.59~1.41, I2 =0%).” | Adverse reactions of inactivated virus vaccines, RNA vaccines, and adenovirus vector vaccines were higher in the vaccine group than in the placebo group, OR: 2.97, 95%CI: 1.49~5.92, I_2_ =100%. |
| De Souza *et al.,* 2021 (34) | CoronaVac | Case series | Brazil | Adults | 1 month | Attack Rate in vaccinated with Astrazeneca 9/23 and in unvaccinated 1/3 with a single dose. With two doses of Sinovac 7/43 and without vaccination 1/9 | Not evaluated | We found that 95.5% (21 of 22) and 83.3% (35 of 42) of people who received a single dose of ChAdOx1 or two doses of CoronaVac, respectively, tested positive for IgG antibody (S- RBD) (cutoff point ≥ 50.0 AU/mL). In addition, 25% (3/12) of the unvaccinated patients were IgG (S-RBD) positive, one was PCR positive, and two were PCR negative. Patients infected with SARS-CoV-2 and immunized with ChAdOx1 had the highest median IgG antibody titer against spike protein (6687 AU/mL), which was statistically different compared to IgG levels (S-RBD) of unvaccinated individuals (p-value = 0.0165 and 0.00237 for PCR-positive and-negative respectively). On the other hand, immunoglobulin antibody levels detected against the SARS-CoV-2 nucleocapsid protein in 18.2% (4 of 22) people vaccinated with the ChAdOx1 vaccine indicated recent SARS-CoV-2 infection because this protein is not expressed from this vaccine. |
| Lu et al., 2022 (35) | CoronaVac | Cohort study | China | Patients with epilepsy or neuropsychiatric disorders | 1 month | Not evaluated | “19 people with epilepsy reported an increase in seizure frequency. No episode of status epilepticus or prolonged seizures was reported. Two controls had their first-ever seizure, which was unlikely related to the vaccine”. | Incidence of vaccine side effects similar to that in healthy controls. |
| Dinc et al., 2022 (36) | CoronaVac | Cohort study | Turkey | Healthcare workers | 2 months | “The total antibody positivity after the second dose was 99.4% (98.6-100), which is significantly higher in those with previous SARS-Cov-2 infection”. | Not evaluated | The total antibody positivity after the second dose was 99.4% (98.6-100), which is significantly higher in those with previous SARS-Cov-2 infection. |
| Benjamanukul et al., 2022 (37) | CoronaVac | Cohort study | Thailand | Healthcare workers | 6 months | “Total antibodies against RDB seroconversion 67% (Geometric mean 1.98 U/ml) after the first dose. After the second dose 100% (92.9 U/ml). Levels comparable to sera from convalescent patients but decrease rapidly after 3 months”. | Not evaluated | After the first dose, total antibodies against RDB seroconversion was 67% (Geometric mean 1.98 U/ml). After the second dose, 100% (92.9 U/ml). Levels comparable to sera from convalescent patients but that decrease rapidly after three months. |
| Al-Ali *et al.,* 2022 (38) | CoronaVac, Pfizer, J&J, Moderna and AstraZeneca. | Systematic literature review | Qatar | Adults | 1 month | Not evaluated | “A total of two cases experienced of cardiovascular complications (CV) and haematological events following receiving the CoronaVac vaccine (one Kounis Syndrome, Type I variant and 1 haemophagocytic lymphohistiocytosis).” | After applying the CoronaVac vaccine, 2 cases out of 122 showed cardiovascular or hematological events. |
| Zeng et al., 2022 (39) | CoronaVac | Parallel clinical trial | China | Adults | 8 months | “Seropositivity rates in all vaccination groups in cohorts 1a-14d-2m and 2a-28d-2m were greater than 95% at 28 days after three doses”. | No adverse events are mentioned in the study, however they refer to: “No serious adverse event in either trial was considered by the investigators to be related to vaccination, and no prespecified trial-halting rules were met”. | A third dose of CoronaVac administered two months after the second dose moderately increased the neutralizing antibody levels induced by the first two doses. In the 3 μg group, the GMT in cohort 1a-14d-2m on day 28 after the second dose was 21.8 (95%CI 17.3–27.6), and on day 28 after the third dose was 45.8 (35.7–58.9), and in cohort 2a-28d-2m GMT on day 28 after the second dose was 38.1 (95% CI 28.4–51.1) and on day 28 after the third dose it was 49.7 (39·9–61·9). The GMIs for neutralizing antibodies from baseline to 28 days after the third dose were 22.9 (95%CI: 17.8–29.4) for cohort 1a-14d-2m and 24.8 (19.9 –31.0) for cohort 2a-28d-2m. Seropositivity rates in all vaccination groups in cohorts 1a-14d-2m and 2a-28d-2m were greater than 95% at 28 days after three doses. |
| Rerknimitr et al., 2022 (40) | CoronaVac and AstraZeneca | Cohort study | Thailand | Healthcare workers | 2 months | Not evaluated | “The incidence of cutaneous adverse reactions was 0.94% with the first dose of Sinovac and 1% with AstraZeneca”. | The incidence of cutaneous adverse reactions was 0.94% with the first dose of Sinovac and 1% with AstraZeneca. |
| Oskay et al., 2022 (41) | CoronaVac | Case report | Turkey | Adults older than 50 years | 1 month | Not evaluated | “A 77-year-old male who developed eruptive skin lesions  was admitted to our clinic. Two weeks after receiving the  third injection of the vaccine”. | Leukocytoclastic vasculitis |
| Sritipsukho et al., 2022 (42) | CoronaVac | Cases and controls | Thailand | Adults | 3 months | “The adjusted vaccine effectiveness (VE) was the highest among people who received two doses of CoronaVac plus a boost of BNT162b2 (98%; 95%CI 87-100), followed by those who received two doses of CoronaVac plus a boost of ChAdOx1 nCoV-19 (86%; 95%CI 74-93), two doses of ChAdOx1 nCoV-19 (83%; 95%CI 70-90), one dose of CoronaVac, and one dose of ChAdOx1 nCoV-19 (74%; 95%CI 43-88) and two-dose CoronaVac (60%; 95%CI 49-69). One dose of CoronaVac or ChAdOx1 nCoV-19 had a VE of less than 50%”. | Not evaluated | The adjusted vaccine effectiveness was the highest among people who received two doses of CoronaVac plus a boost of BNT162b2 (98%; 95%CI 87-100), followed by those who received two doses of CoronaVac plus a boost of ChAdOx1 nCoV-19 (86%; 95%CI 74-93), two doses of ChAdOx1 nCoV-19 (83%; 95%CI 70-90), one dose of CoronaVac, and one dose of ChAdOx1 nCoV-19 (74%; 95%CI 43-88) and two-dose CoronaVac (60%; 95%CI 49-69). One dose of CoronaVac or ChAdOx1 nCoV-19 had a VE of less than 50%. Our study demonstrated that vaccine effectiveness increases with the increase in the number of doses of vaccines received. |
| Cucunawangsih *et al.,* 2022 (43) | CoronaVac and Moderna | Cohort study | Indonesia | Adults | 6 months | “The Moderna vaccine booster - mRNA-1273 was administered 6 months after completing the primary vaccination with CoronaVac. After the third dose, the level of anti-S antibodies increased significantly, from a median of 41.7 U/mL (interquartile range [IQR], 22.4-92.5) to 28,394 U/mL (IQR, 20 837-41 646) (p <0.0001). After the third dose, seropositivity with an anti-S antibody level >210 U/mL was observed in all healthcare workers. Age was negatively associated with the level of anti-S antibodies after mRNA-1273 boost”. | “The most common adverse event of the SARS-CoV-2 vaccination was localized pain at the injection site during the first dose, with 25 (45%) reports, and the booster dose with 34 (67%) reports. Then it was followed by malaise, in the first dose with 20 (36%) reports and in the booster dose with 21 (41%) reports”. | The most common adverse event of the SARS-CoV-2 vaccination was localized pain at the injection site during the first dose, with 25 (45%) reports, and the booster dose with 34 (67%) reports. Then it was followed by malaise, in the first dose with 20 (36%) reports and in the booster dose with 21 (41%) reports. |
| Shao *et al.,* 2022 (44) | CoronaVac and Pfizer | Systematic literature review | China | Unspecified | 6 months | “We found full vaccination provided strong protection against each clinical outcome with summary VE ranging from 86.8% to 96.0% Alpha, moderate protection against infection caused by Beta, Gamma and Delta with summary VE ranging from 70.9% to 72.8%, strong protection against severe disease caused by Delta with summary VE ranging from 84.9% to 90.3%, limited protection with summary VE of 23.5% (95% CI, 17.0-29.5) against infection and moderate protection with summary VE ranging from 56.5% to 82.4% against severe diseases caused by Omicron”. | Not evaluated | Vaccination was found to provide strong protection against each clinical outcome (infection, hospitalization, and death) with summary vaccine effectiveness ranging from 86.8% to 96.0% Alpha, moderate protection against Beta, Gamma, and Delta infections with summary vaccine effectiveness ranging from 70.9% to 72.8%, strong protection against severe Delta disease with summary vaccine effectiveness ranging from 84.9% to  90.3 %, limited protection with summary vaccine effectiveness of 23.5% (95%CI, 17.0–29.5) against infection, and moderate  protection with summary vaccine effectiveness ranging from 56.5% to 82.4% against severe Omicron disease. |
| Chen et al., 2022 (45) | CoronaVac | Cohort study | China | Adults | 6 months | “The four variants showed similar trends with much lower GMTs ( p < 0.0001). The maximum GMTs of the four variants were reached on day 21, 52.5, 78.4, 46.0 and 24.0, respectively. Three months after the booster injection, GMTs decreased significantly to 20.4, 37.5, 22.9, and 6.6; seropositivity rates decreased to 80.6%, 92.9%, 83.9% and 42.6% for the Beta, Gamma, Delta and Omicron variants, respectively. Among the four variants, the GMT of the Gamma variant was the highest ( p < 0.0001) and the GMT of the Omicron variant was the lowest ( p < 0.0001). No significant differences in GMTs were observed between the Beta and Delta variants (p > 0.9999)”. | Not evaluated | There was a significant difference between men and women for IgM during the previous two weeks and between three age groups for IgG during the second and third week after vaccination. The GMT level of IgG in the population vaccinated with the COVID-19 vaccine remained at a high level within 25 weeks. It peaked at day 13, indicating that IgG could exist for a longer period exhibiting a positive defense effect against SARS-CoV-2. |
| Lau et al., 2022 (46) | CoronaVac | Systematic literature review | Multicenter | Adults | 1 month | Not evaluated | “AD26.COV2.S vaccine generated a serious adverse event, high fever 6 hours after vaccination. In others, local reactions (such as redness, pain, and swelling) and systemic reactions (such as fatigue, fever, and headache) were frequently observed” | AD26.COV2.S vaccine generated a serious adverse event, high fever 6 hours after vaccination. In others, local reactions (such as redness, pain, and swelling) and systemic reactions (such as fatigue, fever, and headache) were frequently observed. Higher rates of serious adverse events were noted in Ad5-vectored COVID-19, AD26.COV2.S, ChAdOx nCoV-19, and mRNA-1273. There was no mortality documented for any vaccine. Antispike IgG and neutralizing antibodies were reported in 13 of 19 studies with seroconversion changes. |
| Mok *et al.,* 2022 (47) | CoronaVac and BNT162b2 | Cohort study | China | Adults | 1 month | “The geometric mean PRNT50 titers in those vaccinated with the BNT162b2 and CoronaVac vaccines were 251.6 and 69.45, while the PRNT90 titers were 98.91 and 16.57, respectively”. | Not evaluated | The geometric mean PRNT_50_ titers in those vaccinated with the BNT162b2 and CoronaVac vaccines were 251.6 and 69.45, while the PRNT_90_ titers were 98.91 and 16.57, respectively. All those vaccinated with BNT162b2 and 45 (91.8%) out of 49 vaccinated with CoronaVac achieved the 50% protection threshold for PRNT_90_. |
| Riad et al., 2021 (48) | CoronaVac | Cross sectional | Turkey | Healthcare workers | 1 month | Not evaluated | “62.5% of them experienced at least one side effects (SE). Injection site pain (41.5%) was the most common local SE, while fatigue (23.6%), headache (18.7%), muscle pain (11.2%) and joint pain (5.9%) were the common systemic SEs. Female healthcare workers (67.9%) were significantly more affected by local and systemic SEs than male colleagues (51.4%)”. | Description in percentages of side effects per vaccine: pain at the injection site (41.5%), fatigue (23.6%), headache (18.7%). These were the most common. |
| Chen *et al.,* 2022 (49) | CoronaVac | Systematic literature review | China | Healthcare workers (HCWs), organ transplant recipients, and patients with specific diseases. | 1 month | “The studies showed that the mRNA vaccine had the highest efficacy (94.5 to 95%), followed by viral vector vaccines (66.7 to 91.6%) and inactivated vaccines (72.8 to 83,5 %)”. | “This study found that the safety of SARSCoV-2 vaccines was acceptable, that most adverse events were mild and transient, and that the incidence of systemic adverse events was low”. | The vaccines evaluated in the articles studied were grouped into 1. Viral vector vaccine, 2. mRNA vaccine, and 3. Inactivated vaccine. The studies showed that the mRNA vaccine had the highest efficacy (94.5 to 95%), followed by viral vector vaccines (66.7 to 91.6%) and inactivated vaccines (72.8 to 83,5 %). This study found that the safety of SARSCoV-2 vaccines was acceptable, that most adverse events were mild and transient, and that the incidence of systemic adverse events was low. Compared with the unvaccinated or placebo group, vaccination with the viral vector vaccine may increase the risk of thrombosis but decrease the risk of death**.** |
| Calil *et al.,* 2021 (50) | CoronaVac | Cohort study | Brazil | Pregnant women | 4 months | “Ten mothers had specific IgA antibody levels above the seroconversion value at week 7 (21 days after the second dose)”. | “No significant adverse reactions were reported in either the mothers or their babies”. | No significant adverse reactions were reported in either the mothers or their babies. Ten mothers had specific IgA antibody levels above the seroconversion value at week 7 (21 days after the second dose). Among the ten mothers who donated a sample four months after the first dose, five still had specific IgA levels above the seroconversion value at that time. In our series, four mothers had COVID-19, of which three had elevated levels of anti-SARS-CoV-2 IgA antibodies in W0. One of them donated her milk four months after the first dose of the vaccine, and it still had high levels of specific IgA (anti-SARS-CoV-2 specific IgA ratio = 4.0). |
| Clavero *et al.,* 2022 (51) | CoronaVac and BNT162b2 | Cohort study | Chile | Patients with chronic hemodialysis | 16 months | “Patients who received the BNT162b2 vaccine had a higher humoral response compared to those who received the CoronaVac vaccine (4 months after the second dose: BNT162b2: 88.89%, CoronaVac: 51.97%, p < 0.001; 4 months after the booster: BNT162b2: 98.77%, CoronaVac: 86.61%, p < 0.001)”. | Not evaluated | Patients who received the BNT162b2 vaccine had a higher humoral response compared to those who received the CoronaVac vaccine (4 months after the second dose: BNT162b2: 88.89%, CoronaVac: 51.97%, p < 0.001; 4 months after the booster: BNT162b2: 98.77%, CoronaVac: 86.61%, p < 0.001). |
| Kara *et al.,* 2022 (52) | CoronaVac | Cohort study | Turkey | Adults | 1 month | “The correlation analysis in the non-prior infection study arm (in patients with severe obesity and normal weight controls) demonstrated that age (p = 0.018, r = − 0.211) and BMI (p = 0.008, r: − 0.237) were inversely correlated with SARS-CoV-2 IgG titers in individuals vaccinated with BNT162b2; age (p = < 0.001, r = − 0.415), BMI (p = < 0.001, r = − 0.431), T2DM (p = 0.007, r = − 0.232), and HT (p = < 0.001, r: − 0.429) were inversely correlated with SARS-CoV-2 IgG titers in individuals vaccinated with CoronaVac”. | Not evaluated | The correlation analysis in the non-prior infection study arm (in patients with severe obesity and normal weight controls) demonstrated that age (p = 0.018, r = − 0.211) and BMI (p = 0.008, r: − 0.237) were inversely correlated with SARS-CoV-2 IgG titers in individuals vaccinated with BNT162b2; age (p = < 0.001, r = − 0.415), BMI (p = < 0.001, r = − 0.431), T2DM (p = 0.007, r = − 0.232), and HT (p = < 0.001, r: − 0.429) were inversely correlated with SARS-CoV-2 IgG titers in individuals vaccinated with CoronaVac. |
| Chui *et al.,* 2022 (53) | CoronaVac and BNT162b2 | Case series | China | Patients with incident thromboembolic events or hemorrhagic stroke event | 1 month | No evaluated | “CoronaVac: 402 thromboembolic events and 49 hemorrhagic stroke events, Pfizer/BNT162b2 mRNA: 334 thromboembolic events and 57 hemorrhagic stroke events”. | CoronaVac: 402 thromboembolic events and 49 hemorrhagic stroke events, Pfizer/BNT162b2 mRNA: 334 thromboembolic events and 57 hemorrhagic stroke events.  An increased risk of hemorrhagic stroke was detected in those vaccinated with BNT162b2 14-27 days after the first dose with an adjusted IRR of 2.53 (95% CI 1.48-4.34) and 0-13 days after the second dose with an adjusted IRR of 2.69 (95% CI 1.54-4.69). No statistically significant risk of thromboembolic events was observed for either vaccine.  The incidence of thromboembolic events or hemorrhagic strokes after vaccination is lower than that of SARS-CoV-2 positive cases. |
| Demirhindi *et al.,* 2022 (54) | CoronaVac and BNT162b2 | Cohort study | Turkey | Healthcare workers | 6 months | “At the end of the 6th month, no antibodies were detected in 16.7% of Cohort-I participants, and anti-S-RDB IgG levels showed a decrease of 60% compared to the levels of the 3rd month. The antibody concentrations of the 6th month were found to have increased by an average of 5.13 times compared to the 3rd-month levels in Cohort-II and 20.4 times in Cohort-III” | “Reactions were predominantly mild (68.3%) or moderate (29.9%), and consisted of injection site pain (88.2%), induration (35.5%), headache (44.4%), and myalgia (43.3%)” | Cohort I (CoronaVac(CV)/CV - N:50): 2 reported COVID-19 after the second dose. Anti-S-RBD-IgG and total anti-spike/anti-nucleocapsid-IgG antibody concentrations were determined.  Cohort I (CoronaVac(CV)/CV - N:50): No antibodies were detected in 16.7% at six months after the second dose, and the level of Anti-S-RBD-IgG decreased by 60% compared to the third month.  Cohort II (CV/CV/CV - N: 17): The antibody concentration increased by an average of 5.13 times compared to the third month.  Cohort III (CV/CV/Pfizer (BNT) - N:168): Anti-S-RBD-IgG was detected in all individuals. Antibody concentration increased 20.4 times compared to the third month. Heterologous CV/CV/BNT vaccination was able to induce a stronger humoral response. Anti-S-RBD IgG levels were significantly higher in the CV/CV/BNT cohort than in the other groups. Cohort II (CV/CV/CV - N:17): 7 reported COVID-19 between the second and third dose. Cohort III (CV/CV/Pfizer(BNT) - N:168): No infection was reported between the second and third doses. |
| Shivari *et al.,* 2022 (55) | CoronaVac | Scoping review | Unspecified | Unspecified | 1 month | Not evaluated | “Cardiovascular diseases (thromboembolic events, myocarditis/pericarditis, Takotsubo cardiomyopathy, arrhythmias, and myocardial infarction) have been reported in individuals after vaccination against COVID-19” | Cardiovascular diseases (thromboembolic events, myocarditis/pericarditis, Takotsubo cardiomyopathy, arrhythmias, and myocardial infarction) have been reported in individuals after vaccination against COVID-19; however, for most reports of complications, the association between vaccination and complications has not been demonstrated.  These events are rare and do not affect the multicenter risk/benefit evaluation of COVID-19 vaccines. More longitudinal studies with control groups are needed to portray a more obvious picture of the issue. |
| Fu et al., 2021 (56) | CoronaVac | Cohort study | China | Adults | 1.5 months | “The results  showed that 62.5% and 75% of the participants achieved neutralizing antibody seroconversion on  D14 and D42, respectively” | Not evaluated | Determination of immune response induced by inactivated vaccines (Sinopharm and Sinovac) by analyzing seroconversion of antibodies, 12 key cytokines, and 34 lymphocyte subsets at three-time points (D1 (before first injection), D14 (after first injection) and D42 (equivalent to day 21 after second injection). 62.5% and 75% of the participants achieved neutralizing antibody seroconversion on D14 and D42, respectively. After vaccination, IL-5 and IL-6 increased, and INF-γ decreased. IL6, IL-1B, INF-γ, IL-8, and IL-12p70 showed statistical significance in the comparison of different groups. In terms of lymphocyte subsets, CD3 +, CD56 +, CD3 + CD8 +, CD8 + CD71 +, and CD56 + CD71 + showed upward trend, while CD19 +, CD4 + CD8 +, CD8 + CD45RA +, CD4 + HLA-DR +, CD8 + HLA-DR +, and CD8 + CD38 + showed downward trend. This clinical observation showed that the inactivated vaccines induced both humoral immunity by producing neutralizing antibodies and cellular immunity. The cellular immunity induced by these two vaccines was a Th2-biased response, which may also lead to a mild Th1-type response |
| Pinpathomrat *et al.,* 2022 (57) | CoronaVac | Pilot study | Thailand | Adults | 1.5 months | “The anti-RBD-IgG levels, the neutralising function  against delta variants, and T cell responses were significantly increased after boosting via both routes” | “Immediate and delayed local reactions were frequently observed in the fractional intradermal boost, but systemic side effects were  significantly decreased compared to the conventional intramuscular boost.” | Immediate and delayed local reactions were frequently observed in the fractional intradermal booster, but systemic side effects were significantly decreased compared to the conventional intramuscular booster. The anti-RBD-IgG levels, the neutralizing function against Delta variants, and T-cell responses were significantly increased after boosting via both routes. Interestingly, the shorter interval elicited higher immunogenicity compared to the extended interval. |
| Saida *et al.,* 2022 (58) | CoronaVac | Case series | Tunisia | Adults | 1 month | Not evaluated | The appearance of thrombotic thrombocytopenic purpura (TTP) post-vaccination. | The appearance of thrombotic thrombocytopenic purpura (TTP) post-vaccination. |
| Chaijaras *et al.,* 2022 (59) | CoronaVac | Case report | Thailand | Adults | 1 month | Not evaluated | Pruritic blanchable erythematous macules and patches and excoriated papules and plaques on the trunk and extremities, which resolved with postinflammatory hyperpigmentation. | Pruritic blanchable erythematous macules and patches and excoriated papules and plaques on the trunk and extremities, which resolved with postinflammatory hyperpigmentation. |
| Oliveira *et al.,* 2022 (60) | CoronaVac | Cohort study | Brazil | Adults | 6 months | “In the vaccinated group, a rapid increase in RBD response and neutralizing antibody levels upon vaccination were observed in individuals with a history of pre-exposure (6–10 months) to SARS-CoV-2 infection compared to naïve individuals. Anti-RBD antibodies were detected in both cohorts, peaking between 45 and 90 days after infection or vaccination, followed by a steady decline over time”. | Not evaluated | RBD-specific and neutralizing antibodies were analyzed. For all analyses, statistical significance was assumed when p < 0.05. Mean and standard deviation were calculated to compare the values obtained from optical density (OD) and viral neutralization titers. In the vaccinated group, a rapid increase in RBD response and neutralizing antibody levels upon vaccination were observed in individuals with a history of pre-exposure (6–10 months) to SARS-CoV-2 infection compared to naïve individuals. Anti-RBD antibodies were detected in both cohorts, peaking between 45 and 90 days after infection or vaccination, followed by a steady decline over time. |
| Chan *et al.,* 2021 (61) | CoronaVac and BNT162b2 | Cross-sectional | China | Adults | 2 months | “In 64% of the subjects, the neutralizing effects of NELF persisted for at least 50 days. Moreover, 85% of Comirnaty recipients exhibited S1-specifific IgA and IgG responses in plasma by 14 ± 2 days after the first dose. By 7 ± 2 days after the booster, all plasma samples possessed S1-specifific IgA and IgG responses and were neutralizing. The induction of S1-specifific plasma antibodies by CoronaVac was IgG dominant, and 83% of the subjects possessed S1-specifific IgG by 7 ± 2 days after the booster, with neutralizing effects”. | Not evaluated | Quantification of IgA and IgG specific to SARS-CoV-2 S1 protein by ELISA in NELF and plasma samples. The neutralization effect of these two sample types was evaluated by surrogate ACE-SARS-CoV-2 Spike protein ELISA. Pfizer/BioNTech (Comirnaty): In Nasal Epithelial Lining Fluid (NELF) samples, 72% of subjects became IgA+IgG+, while in 62.5% of subjects, the samples were neutralizing seven days after the second dose. In 45% of the subjects, their NELF remained neutralizing 50 days after the booster of Comirnaty. In plasma, 91% and 100% Comirnaty subjects possessed S1-specific IgA+IgG+ 14 days after the first dose and 7 days after booster, respectively. The plasma collected 7 days after the booster was 100% neutralizing. The induction of S1-specific antibody by CoronaVac was IgG dominant, and 70% of the subjects possessed S1-specific IgG 7 days after booster and were all neutralizing. |
| Dadras *et al.,* 2022 (62) | CoronaVac | Systematic literature review | Iran | Adults | 1 month | Not evaluated | “The most common local side effects  included injection site pain and swelling, redness, and pruritus”. | The most common local side effects included pain and swelling at the injection site, redness, and itching. Meanwhile, fatigue, headache, muscle pain, fever, and gastrointestinal symptoms, including abdominal pain and diarrhea, were among the most common systemic adverse effects. |
| Navarro-Olivos *et al.,* 2022 (63) | CoronaVac and BNT162b2 | Cohort study | United States | Healthcare workers | 6 months | “The differences observed in the levels of anti-Spike IgG antibodies between people with and without previous SARS-CoV-2 infection were not statistically significant.” | “25.4% were positive for SARS-CoV-2 before vaccination. Only one person did not react to the two doses of the vaccine. Most self-reported reactions stopped within a short period - less than three days”. | 25.4% were positive for SARS-CoV-2 before vaccination. Only one person did not react to the two doses of the vaccine. Most self-reported reactions ceased in less than three days. The differences observed in terms of the levels of IgG anti-Spike antibodies between people with and without previous SARS-CoV-2 infection were not statistically significant. |
| Mckeon *et al.,* 2022 (64) | CoronaVac, Pfizer-BioNTech, Moderna and Oxford-AstraZeneca | Cohort study | United States | Adults | 12 months | “After a COVID-19 diagnosis, vaccinated patients were significantly less likely than unvaccinated patients to be hospitalized” | Not evaluated | The risk of being diagnosed with COVID-19 post-vaccination became progressively lower during the study period (hazard ratio and 95% confidence interval for BNT162b2 was 0.21 [0.13, 0.35] and for mRNA-1273 was 0.27 [0.17, 0.42] for days ≥43). After a COVID-19 diagnosis, vaccinated patients were significantly less likely than unvaccinated patients to be hospitalized (for BNT162b2, 28.0% vs.. 43.4%; for mRNA-1273, 37.2% vs. 45.6%) and significantly less likely to die (for BNT162b2, 4.0% vs. 12.1%; for mRNA-1273, 5.6% vs. 14.5%). Antibodies were detected in 98.1% (309/315) and 96.0% (308/321) of BNT162b2 and mRNA-1273 patients, respectively. |
| Han *et al.,* 2022 (65) | CoronaVac | Cross sectional | China | Immunosuppressed | 2 months | “Neutralizing antibodies against the D614G variant were detected in 74.5% (35/47) of HIV-infected patients, and neutralizing antibodies against the Delta variant in 66.0% (31/47) of patients.” | “A total of 19.1% (9/47) of HIV-infected patients had adverse reactions within 28 days of full vaccination, and the majority occurred within seven days of full vaccination.” | A total of 19.1% (9/47) of HIV-infected patients had adverse reactions within 28 days of full vaccination, and the majority occurred within seven days of full vaccination. The most common local event was pain at the injection site (6.4%), which generally subsided within 24 hours. Fatigue (6.4%), headache (6.4%), and fever (6.4%) were common systemic adverse events after vaccination. Neutralizing antibodies against the D614G variant were detected in 74.5% (35/47) of HIV-infected patients, and neutralizing antibodies against the Delta variant in 66.0% (31/47) of patients. The GMT for the Delta variant (14, 95% CI 11–19) was 45% that of the D614G variant (31, 95% CI 20–47) (p=0.002). |
| Díaz *et al.,* 2022 (66) | CoronaVac, BNT162b2 and Vaxzevria | Cohort study | Chile | Adults | 1 month | “Among patients with cirrhosis, vaccination against COVID‐19 was associated with a lower rate of hospitalization than individuals with no vaccination.” | Not evaluated | Patients with COVID‐19 and underlying cirrhosis frequently required hospitalization during the infection (42.9% vs. 7.7% in the general population). Among patients with cirrhosis, vaccination against COVID‐19 was associated with a lower rate of hospitalization than individuals with no vaccination. |
| Choi *et al.,* 2022  (67) | CoronaVac, BNT162b2 and BBIBP-CorV | Systematic literature review | Multicenter | Children | 2.5 months | “Vaccine effectiveness after two doses against omicron infections was 41·6% (95% CI 28·1–52·6; eight non-randomised studies of interventions [NRSIs]; CoE low), 36·2% (21·5–48·2; six NRSIs; CoE low) against symptomatic COVID-19, 75.3% (68·0–81·0; six NRSIs; CoE moderate) against COVID-19-related hospitalisations, and 78% (48–90, one NRSI; CoE very low) against MIS-C.” | “The risk of solicited local reactions was 2.07 (1·80–2·39; two RCTs; CoE moderate) after one dose and 2.06 (1·70–2·49; two RCTs; CoE moderate) after two doses. The risk of solicited systemic reactions was 1·09 (1·04–1·16; two RCTs; CoE moderate) after one dose and 1.49 (1·34–1·65; two RCTs; CoE moderate) after two doses.” | Vaccines against COVID-19 in children aged 5 -11 years produced a favorable immune response and were effective against COVID-19. |
| Bertola *et al.,* 2022 (68) | CoronaVac | Case series | Turkey | Adults | 1 month | Not evaluated | “SARS-CoV-2 vaccination can lead to subacute thyroiditis as a phenomenon of ASIA syndrome”. | Autoimmune/inflammatory syndrome induced by adjuvants (ASIA syndrome) occurred after vaccination. |
| Ozdede *et al.,* 2022 (69) | CoronaVac and BNT162b2 | Cross-sectional | United States | Adults | 1 month | “BionNTech ensured significantly better efficiency than CoronaVac against COVID-19 in all patient groups” | “The adverse events were more frequent among those vaccinated with BionNTech than those vaccinated with CoronaVac.” | The adverse events were more frequent among those vaccinated with BionNTech than those vaccinated with CoronaVac. BionNTech ensured significantly better efficiency than CoronaVac against COVID-19 in all patient groups (BS 1.4% vs. 10.1%; FMF 3.2% versus 12.2%, RD 2.7% vs. 6.4%). |
| Czarnowska *et al.,* 2022 (70) | CoronaVac, Pfizer-BioNTech, Moderna and Oxford-AstraZeneca | Cohort study | Poland | Adults | 5 months | Not evaluated | “Mild symptoms after immunization, often after the first dose, were reported in 70.6% of individuals. Symptoms included arm pain (47.5% first dose and 38.7% second dose), fever/chills/flu-like symptoms (17.1% first dose and 20.5% second dose), and fatigue (10, 3% first dose and 11.3% second dose). Only one individual presented with severe side effects.” | 82.4% were vaccinated with nucleoside-modified messenger RNA (mRNA) vaccines. Mild symptoms after immunization, often after the first dose, were reported in 70.6% of individuals. Symptoms included arm pain (47.5% first dose and 38.7% second dose), fever/chills/flu-like symptoms (17.1% first dose and 20.5% second dose), and fatigue (10, 3% first dose and 11.3% second dose). Only one individual presented with severe side effects. Nine patients (0.4%) had a SARS-CoV-2 infection confirmed despite vaccination |
| Chen *et al.,* 2022 (71) | CoronaVac, Pfizer-BioNTech, Moderna and Oxford-AstraZeneca | Cohort study | Reino Unido | Adults | 4 months | “The level of anti-SARS-CoV-2 IgG was higher in patients who received mRNA-1273 than in those who received AZD1222” | Not evaluated | The anti-Sars-COV-2 IgG seropositivity rate was 78.8% (89/113) for AZD1222 and 83.1% (108/130) for mRNA-1273. The level of anti-SARS-CoV-2 IgG was higher in patients who received mRNA-1273 than those who received AZD1222 (β: 30.15, 95%CI: 11.67-48.63, p = 0.002). |
| Cox *et al.,* 2021 (72) | CoronaVac, Pfizer-BioNTech, Moderna and Oxford-AstraZeneca | Cohort study | United States | Adults | 1 month | Not evaluated | “Most adverse events occurred after the second dose (33, 59%). The most frequent grade 1 Adverbs Events (AEs) included injection site pain (7,14.6%), fever (7,14.6%), fatigue (7,14.6%), chills (5,10.4%)”. | The vaccines BNT162B2 by Pfizer (96/185, 52%), MRNA-1273 by Moderna (79/185, 43%), and JNJ-78436735 by Johnson & Johnson (10/185, 5%). At least one AE was observed in 35 patients (19%); The total number of AE was 56.48 Grade 1 (86%) and 8 Grade 2 (14%). Most adverse events occurred after the second dose (33, 59%). The most frequent Grade 1 AEs included pain at the injection site (7, 14.6 %), fever (7, 14.6 %), fatigue (7, 14.6 %), and chills (5, 10.4 %). The most frequent Grade 2 was fatigue (3, 37.5%). The therapy was delayed due to AEs in 3 patients (1.6%). |
| Hasseli *et al.,* 2021 (73) | CoronaVac, Pfizer-BioNTech, Moderna and Oxford-AstraZeneca | Cohort study | Alemania | Adults | 1 month | Not evaluated | “The most common side effect was pain at the injection site (71%), followed by fatigue (41%), headache (33%), and arthralgia (24%).” | 67% were vaccinated with Pfizer-Bionntech, and 28% with AstraZeneca. 70% did not change their immunomodulation during the vaccination period. The most common side effect was pain at the injection site (71%), followed by fatigue (41%), headache (33%), and arthralgia (24%). Only 1% of the patients reported allergic reactions. Side effects lasted in median for two days (range 0-99 days), and 15% reported no relevant side effects. Disease flares after the first COVID-19 vaccination, as reported by patients on a scale of 1 to 10, were indicated in 13% of the patients. |
| Murali *et al.,* 2022 (74) | CoronaVac, Pfizer-BioNTech, Moderna and Oxford-AstraZeneca | Cohort study | India | Adults | 3 months | “The vaccine effectiveness against COVID-19 disease in the ≥45 age group was 61.3% (95% CI: 43.6-73.4) at least two weeks after receiving the second dose of Covishield.” | Not evaluated | The coverage of two doses of Covishield in the 18+ and 45+ age groups was 18% and 31%, respectively. The vaccine effectiveness against COVID-19 disease in the ≥45 age group was 61.3% (95% CI: 43.6-73.4) at least two weeks after receiving the second dose of Covishield. |
| Ortiz *et al.,* 2022 (75) | CoronaVac, Pfizer-BioNTech and Oxford-AstraZeneca | Cross-sectional | Ecuador | Adults | 1 month | Not evaluated | “The most reported adverse events were pain or swelling at the injection site 17.2% and headache 13.3%. 87.0% were mild, 11.5% moderate, and 1.5% severe”. | The most reported adverse events were pain or swelling at the injection site 17.2% and headache 13.3%. 87.0% were mild, 11.5% moderate, and 1.5% severe. The most reactogenic vaccine was AstraZeneca, with 57.8%, Pfizer at 24.9%, and Sinovac at 17.3%. |
| Cheng *et al.,* 2021 (76) | CoronaVac, Pfizer-BioNTech and Oxford-AstraZeneca | Systematic literature review | Multicenter | Adults | 1 month | “All vaccines had a good preventive effect on COVID-19 (RR = 0.17, 95% CI: 0.09-0.32), and the mRNA vaccine (RR = 0.05, 95% IC: 0.03-0.09) was the most effective against COVID-19, while the inactivated vaccine (RR = 0.32, 95% CI: 0.19-0.54) was the least” | “The risk of overall adverse events showed an increase in the vaccine group after the first (RR = 1.46, 95% CI: 1.03-2.05) or second (RR = 1.52, 95% CI: 1.04-2.20) injection” | All vaccines had a good preventive effect on COVID-19 (RR = 0.17, 95% CI: 0.09-0.32), and the mRNA vaccine (RR = 0.05, 95% IC: 0.03-0.09) was the most effective against COVID-19, while the inactivated vaccine (RR = 0.32, 95% CI: 0.19-0.54) was the least. Regarding safety, the risk of overall adverse events showed an increase in the vaccine group after the first (RR = 1.46, 95% CI: 1.03-2.05) or second (RR = 1.52, 95% CI: 1.04-2.20) injection. However, compared with the first injection, the risk of local adverse events (RR = 2.64, 95% CI: 1.02-6.83 vs. RR= 2.25, 95%CI: 0.52- 9.75) and systemic adverse events (RR = 1.33, 95%CI: 1.21-1.46 vs. RR = 1.59, 95% CI: 0.84-3.01) decreased after the second injection. |
| Meng *et al.,* 2021 (77) | CoronaVac and Pfizer-BioNTech | Systematic literature review | Multicenter | Children | 1 month | “The RCTs showed that the immune response to BNT162b2 in adolescents aged 12–15 years was  non-inferior to that in young people aged 16–25 years, while with 3 µg CoronaVac injection the  immune response was stronger than with 1.5 µg. The efficacy of BNT162b2 was 100% (95% CI: 75.3 to  100), based on one RCT” | “The investigated COVID-19 vaccines had good safety profiles  in children and adolescents. Injection site pain, fatigue, headache, and chest pain were the most  common adverse events” | The reported security profiles were reported. |
| Cheng *et al.,* 2022 (78) | CoronaVac, Pfizer-BioNTech and Oxford-AstraZeneca | Systematic literature review | Multicenter | Adults | 1 month | “The immunity against COVID-19 after the prime vaccination waned over time, especially in the  populations primed with inactivated vaccines, in which the seropositive rate of antibodies was only  28% (95% CI: 17–40%). Booster vaccination could significantly increase the antibody responses, and  heterologous immunization was more effective than homologous immunization (neutralization titers:  1.65 vs. 1.27; anti-RBD IgG: 1.85 vs. 1.15)” | “Compared with the initial two doses of vaccines, a  booster dose did not induce additional or severe adverse events” | A total of 28 studies, nine combinations of booster vaccinations, and 5870 subjects were included in the meta-analysis. Random effect models were used to estimate pooled immunogenicity and safety. Immunity against COVID-19 after the main vaccine decreased over time, especially in populations vaccinated with inactivated vaccines, in which the seropositive rate of antibodies was only 28% (95% CI: 17-40%). Booster vaccination could significantly increase antibody responses, and heterologous immunization was more effective than homologous immunization (neutralization titers: 1.65 vs. 1.27; anti-RBD IgG: 1.85 vs. 1.15); in particular, the mRNA-inactivated vaccine combination had the highest antibody responses (neutralization titers: MRAW = 3.64, 95% CI: 3.54–3.74; anti-RBD IgG: 3.73, 95% CI: 3.59–3.87). |
| Chua *et al.,* 2022 (79) | CoronaVac, Pfizer-BioNTech, Moderna and Oxford-AstraZeneca | Scoping review | Singapore | Adults | 1 month | Not evaluated | “The females were significantly more likely to have severe clinical presentation and poorer outcomes compared to males. Additionally, viral vector vaccines were the predominant vaccine type administered in early-onset post-COVID-19-vaccination Guillain–Barré syndrome (GBS) and GBS occurring after the 1st vaccination dose. It was also shown that reported cases of post-vaccination GBS generally displayed a positive response to conventional treatment and had favourable posttreatment outcomes.” | Guillain Barré syndrome case description. |
| Chantasrisawad *et al.,* 2022 (80) | CoronaVac | Parallel clinical trial | Thailand | Children | 3 months | “The geometric means (GMs) of surrogate virus neutralization test (sVNT) and pseudovirus neutralization test (pVNT) were 72.2 %inhibition (95 %CI 67.2–77.6) and 499 (95 %CI 399–624), respectively. The proportion of children with sVNT against Omicron strain 68 %inhibition increased from none to 70.2 %. The geometric mean ratio (GMR) of sVNT and pVNT compared with a parallel cohort were 4.3 and 12.2, respectively”. | Not evaluated | Of 59 children: 20 were CoronaVac recipients, and 39 were Covilo recipients. The median interval for the primary series was 49 days (IQR 33–51). After booster, the geometric means (GM) of sVNT and pVNT were 72.2% inhibition (95% CI 67.2–77.6) and 499 (95% CI 399–624), respectively. The proportion of children with sVNT against Omicron strain 68 % inhibition increased from zero to 70.2%. The geometric mean ratios (GMR) of surrogate virus neutralization test - sVNT and pseudovirus neutralization test pVNT compared to a parallel cohort were 4.3 and 12.2, respectively. The GMR of sVNT and pVNT between children who received a booster dose at >6 weeks intervals were 1.2 (95% CI: 1.1–1.3) and 1.8 (95% CI 1.2 to 2.7) compared with a 4-6 weeks interval. |
| Kanokudom *et al.,* 2022 (81) | CoronaVac and Oxford-AstraZeneca | Parallel clinical trial | Thailand | Adults | 2 months | “The third dose significantly produced total Ig anti-RBD GMT of 20,787 U/mL at 14 days post-vaccination (p < 0.0001). Additionally, anti-RBD immunoglobulin is slightly reduced at 28 days to determine the level of anti-RBD IgG.” | “Further analysis revealed that Adverse Events after receiving the BBIBP vaccine were considerably lower than the other two types of vaccines, except for vomiting.” | Further analysis revealed that Adverse Events after receiving the BBIBP vaccine were considerably lower than the other two types of vaccines, except for vomiting. Joint pain was substantial in 40.4% of all the participants who received the AZD1222 vaccine. |
| Abarca *et al.,* 2022 (82) | CoronaVac | Parallel clinical trial | Chile | Adults | 6 months | “Schedules showed a high probability of being free of COVID-19: 96.7% (0-14) and 97.9% (0-28) (non-inferiority p-value < 0.001)” | “A total of 867 local and 1395 systemic solicited adverse events were reported after the second dose” | A total of 867 local and 1395 systemic solicited adverse events were reported after the second dose. These AEs were reported in 31.2% and 32.9% of the vaccinated participants for the 0–14 and 0–28 schedules, respectively. The 0-14 schedules did not show inferiority to the 0-28 schedules (p < 0.0001) in terms of AE frequency. Sinovac is safe, especially in participants ≥60 years. A total of 34 and 24 cases of COVID-19 were registered in the 0-14 and 0-28 schedules, respectively (p = 0.083). Both schedules showed a high probability of being free of COVID-19: 96.7% (0-14) and 97.9% (0-28) (non-inferiority p-value < 0.001). A Kaplan–Meier analysis showed that a probability of 0.98 for being free of COVID-19- was achieved at day 91 for the 0–14 schedule and at day 133 for the 0–28 schedule. |
| Boongird *et al.,* 2022 (83) | CoronaVac | Cohort study | Thailand | Adults | 6 months | “At six months, median anti-RBD IgG titers (IQR) decreased significantly from baseline on HD (1741 (1136–3083) BAU/mL vs. 373 (188–607) BAU/mL) and PD (1093 (617–1911)) BAU/mL vs. 180 (126–320) BAU/mL) groups, as did the mean percent inhibition of neutralizing antibodies (HD: 96% vs. 81%; PD: 95% vs. 73%) (all p<0.01)” | Not evaluated | At six months, median anti-RBD IgG titers (IQR) decreased significantly from baseline on HD (1741 (1136–3083) BAU/mL vs. 373 (188–607) BAU/mL) and PD (1093 (617–1911)) BAU/mL vs. 180 (126–320) BAU/mL) groups, as did the mean percent inhibition of neutralizing antibodies (HD: 96% vs. 81%; PD: 95% vs. 73%) (all p<0.01). Age and the intensity of the post-vaccination serological response were predictors of early loss of humoral seroprotection. By contrast, cell-mediated immunity remained unchanged. |
| Kang *et al.,* 2022 (84) | CoronaVac | Cohort study | China | Adults | Not reported | Prevention of infection, symptomatic infection, pneumonia and severe or critical illness | Not evaluated | Of 10,805 participants, 1.3% contracted infections, 1.2% developed symptomatic infections, 1.1% had pneumonia, and 0.2% had severe or critical illness. Adjusted Vaccine Effectiveness Scores (VE) of full vaccination were 51.8% (95% CI, 20.3% to 83.2%) against infection, 60.4% (CI, 31.8% to 88.9%) against symptomatic infection and 78.4% (CI, 56.9% to 99.9%) against pneumonia. In addition, full vaccination was 100% (CI, 98.4% to 100.0%) effective against severe or critical illness. In contrast, the adjusted VEs of partial vaccination against infection, symptomatic infection, and pneumonia were 10.7% (CI, -41.2% to 62.6%), 6.8% (CI, -47.4% to 61.0%) and 11.6% (CI, -42.6% to 65.8%), respectively. |
| Cheng *et al.,* 2022 (85) | CoronaVac | Cohort study | China | Adults | 1 month | Antibody levels | Not evaluated | Concerning the neutralization of the WT virus (SARS-COV2 virus), those vaccinated with Pfizer had reductions of 4.8, 3.4, 4.6, 11.3, and 15.5 times in the geometric mean of the titers of antibodies (GMT) to BA.1, BA.2, BA.2.12.1, BA. 4 and BA.5 virus, respectively. Similarly, those vaccinated with CoronaVac (Sinovac) had 8.0, 7.0, 11.8, 12.0, and 12.0-fold GMT reductions, and those vaccinated with two doses of CoronaVac BNT162b2-boosted had 6.1, 6.7, 6.3, 13.0 and 21.2 fold GMT reductions, respectively. Vaccinated people with BA.2 breakthrough infections had higher GMT antibody levels against BA.4 (36.9) and BA.5 (36.9) than unvaccinated people with BA.2 infections (BA.4 GMT 8.2; BA.5 GMT 11.0). |
| Shenyu *et al.,* 2022 (86) | CoronaVac | Parallel clinical trial | China | Adults | 3 months | Antibody levels | Adverse events post-vaccination | Overall, 480 participants were enrolled, with 120, 120, and 240 randomly assigned to groups C1, C2, and S, respectively. As the lower bound of the two-sided 95% confidence interval (CI) of the difference for the antibody seroconversion rate against SARS-CoV-2 was greater than -10%, the immune response to CoronaVac in group C (93 .1% [89.0, 96.0]) was non-inferior to that of group S (95.2% [91.5, 97.6]) in the per-protocol pool-set. A lower GMT of antibodies against SARS-CoV-2 was observed in group C compared to group S (27.5 vs. 38.1, P = 0.0001). Decreased immune response to CoronaVac was observed primarily in participants who received IIV4 concomitantly with their second dose of CoronaVac (C2 subgroup), with a seroconversion rate of 89.7% (95% CI: 82.6-94.5 %) and a GMT of 23.3. |
| Li *et al.,* 2022 (87) | CoronaVac | Parallel clinical trial | China | Adults | 9 months | Antibody levels | CoronaVac | Adverse reactions were reported by 26 (19%) participants in the low-dose group and 33 (24%) in the high-dose group within 14 days of booster vaccination, significantly fewer than the 54 (39%) participants in the CoronaVac group (p<0 0001). |
| Demir *et al.,* 2022 (88) | CoronaVac | Cases and controls | Turkey | Adults | 6 months | all-cause mortality, hospitalization, intensive care unit admission, acute kidney injury, cytokine storm, and acute respiratory distress syndrome | Not evaluated | Four patients in the vaccinated group (4.9%) and nine patients (11%) in the control group died during follow-up (p = 0.247). Seventeen patients (20.7%) in the vaccinated group and thirty-four participants (41.5%) in the control group were hospitalized (p = 0.004). In addition, five vaccinated patients (6.1%) and ten unvaccinated patients (12.2%) were followed up in the ICU during follow-up (p = 0.168). Five vaccinated (6.1%) and ten unvaccinated (12.2%) patients received mechanical ventilation (p = 0.168). In addition, one vaccinated patient died of multiple organ failure secondary to cytokine storm, and three were lost due to respiratory failure. Two of the deceased patients received two doses of CoronaVac, one received two doses of BNT162b2, and one was vaccinated with two doses of CoronaVac and one dose of BNT162b2. No adverse events occurred, and no drug interactions were detected during follow-up. |
| Dheir *et al.,* 2022 (89) | CoronaVac | Cohort study | Turkey | Adults | 6 months | Antibody levels | Not evaluated | Anti-Spike antibodies were measured 28 days after each vaccine dose, 3 and 6 months after the first dose, and compared them between cohorts. After two doses, an anti-spike immunoglobulin G of ≥50 AU/mL was present in hemodialysis (HD), kidney transplant recipients (KTR), and healthy control groups (HG) as 44%, 7.2%, and 58.5%, respectively (p<0.001). Furthermore, the proportion of antibody titers peaked at 86.5%, 23%, and 97.6% (p < 0.001) at month three and decreased significantly at month six in most HD and HG participants, while this effect was not observed in KTRs from basal to month 6 (p < 0.001). During follow-up, the incidence of coronavirus disease 2019 was higher (p < 0.003) in the KTRs compared to the other groups, but there was no need for an intensive care unit, and no deaths were recorded. We found a negative correlation between antibody seroconversion and age (p < 0.016). |
| Cheng *et al.,* 2022 (90) | CoronaVac | Cohort study | China | Adults | Not reported | Prevention of infection, hospital admissions and death | Not evaluated | The incidence rates of COVID infection in the BNT162b2 group (25.58 [95% confidence interval {CI}: 24.63–26.56] per 100 person-years) and the CoronaVac group (38.07 [95% CI: 37.02–39.13] per 100 person-years) were lower than those in the unvaccinated group (45.55 [95% CI: 44.28–46.84] per 100 person-years). Vaccine effectiveness was estimated to be 38% (95% CI: 34–41%) for BNT162b2 and 4% (95% CI: 0–8%) for CoronaVac compared to the unvaccinated group. Unlike the COVID-19 infection, both vaccines demonstrated a reduction in COVID-19-related hospitalizations and deaths. For BNT162b2 recipients, the vaccine effectiveness was 64% (95% CI: 57–69%) for COVID-19-related hospitalization and 86% (95% CI: 80–90%) for death related to COVID-19. For CoronaVac, vaccine effectiveness was 44% (95% CI: 37%–49%) and 70% (95% CI: 64%–75%) for COVID-19-related hospitalization and death, respectively. |
| Camacho *et al.,* 2022 (91) | CoronaVac | Cross-sectional | México | Adults | Not reported | Not evaluated | Adverse events post-vaccination | The prevalence of at least one side effect varied between vaccines and by the number of doses. At dose 1, ChAdOx1 was the vaccine with the highest rate of at least one side effect (85%), followed by Gam-COVID-Vac (80%). Both were associated with greater extension (adjusted OR 2.53, 95% CI 2.16, 2.96 and adjusted OR 2.41, 95% CI 1.76, 3.29, respectively) and severity of side effects (adjusted OR 4.32, 95% CI 3.73, 5.00 and adjusted OR 3.00, 95% CI 2.28, 3.94, respectively). Young age (<50 years), female sex, comorbidity, and a history of allergies were associated with greater extension and severity, regardless of the type of vaccine and possible confounding factors. At dose 2, mRNA-1273 was the vaccine with the highest rate of side effects (88%) and the only vaccine associated with a greater extension (adjusted OR 2.88, 95% CI 1.59, 5. 21) and the severity of symptoms (adjusted OR 3.14, 95% CI 1.82, 5.43). |
| Nguyen *et al.,* 2022 (92) | CoronaVac | Systematic literature review | United States | Adults | Not reported | Not evaluated | Adverse events post-vaccination | Thirty articles were selected (22 case reports, 3 case series, 2 cohort studies, 2 cross-sectional studies, and one clinical trial) encompassing 2020 patients (mean age 53.4 ± 19.9, 91.3% women) describing 93 patients who developed post‐vaccination cutaneous symptoms of connective tissue diseases. |
| Cucunawangsih *et al.,* 2022 (93) | CoronaVac | Cohort study | Indonesia | Adults | 6 months | Antibody levels | Not evaluated | The Moderna - mRNA-1273 vaccine booster was administered six months after completion of primary vaccination with CoronaVac. After the third dose, the level of anti-S antibodies significantly increased, from a median of 41.7 U/mL (interquartile range [IQR], 22.4-92.5) to 28,394 U/mL (IQR, 20,837-41,646) (p <0.0001). After the third dose, seropositivity with an anti-S antibody level >210 U/mL was observed in all healthcare workers. Age was negatively associated with the level of anti-S antibodies after the mRNA-1273 booster. |

**REFERENCES**

1. Peters M, Godfrey C, McInerney P, Munn Z, Trico A, Khalil H. Chapter 11: Scoping Reviews. En: JBI Manual for Evidence Synthesis [Internet]. JBI; 2020. Disponible en: https://wiki.jbi.global/display/MANUAL/Chapter+11%3A+Scoping+reviews

2. Paixao ES, Wong KLM, Alves FJO, de Araújo Oliveira V, Cerqueira-Silva T, Júnior JB, et al. CoronaVac vaccine is effective in preventing symptomatic and severe COVID-19 in pregnant women in Brazil: a test-negative case-control study. BMC Medicine. 2022;20(1):1-8.

3. Deng J, Ma Y, Liu Q, Du M, Liu M, Liu J. Comparison of the Effectiveness and Safety of Heterologous Booster Doses with Homologous Booster Doses for SARS-CoV-2 Vaccines: A Systematic Review and Meta-Analysis. International Journal of Environmental Research and Public Health. 2022;19(17):1-17.

4. Chen W, Zhang L, Li J, Bai S, Wang Y, Zhang B, et al. The kinetics of IgG subclasses and contributions to neutralizing activity against SARS-CoV-2 wild-type strain and variants in healthy adults immunized with inactivated vaccine. Immunology. 2022;167(2):221-32.

5. Liu X, Li Y, Wang Z, Cao S, Huang W, Yuan L, et al. Safety and superior immunogenicity of heterologous boosting with an RBD-based SARS-CoV-2 mRNA vaccine in Chinese adults. Cell Research. 2022;32(8):777-80.

6. Oskay T, Isık M. Leukocytoclastic vasculitis after the third dose of CoronaVac vaccination. Clinical Rheumatology. 2022;41(6):1931-3.

7. Kouhpayeh H, Ansari H. Adverse events following COVID-19 vaccination : A systematic review and meta-analysis. International Inmunopharmacology. 2020;109.

8. Li T, Song R, Wang J, Zhang J, Cai H, He H, et al. Safety and immunogenicity of inactivated SARS-CoV-2 vaccines in people with gastrointestinal cancer. International Journal of Infectious Diseases. 2022;122:874-84.

9. Kuloğlu ZE, El R, Guney-Esken G, Tok Y, Talay ZG, Barlas T, et al. Effect of BTN162b2 and CoronaVac boosters on humoral and cellular immunity of individuals previously fully vaccinated with CoronaVac against SARS-CoV-2: A longitudinal study. Allergy: European Journal of Allergy and Clinical Immunology. 2022;77(8):2459-67.

10. Cristelli MP, Nakamura MR, Viana LA, Tedesco-Silva H, Medina-Pestana J. The Fourth Dose of CoronaVac Vaccine Results in a Small Increase of Seroconversion and Antibody Values among Kidney Transplant Recipients. Transplantation. 2022;106(9):E420-1.

11. Mohammed I, Nauman A, Paul P, Ganesan S, Chen KH, Jalil SMS, et al. The efficacy and effectiveness of the COVID-19 vaccines in reducing infection, severity, hospitalization, and mortality: a systematic review. Human Vaccines and Immunotherapeutics. 2022;18(1).

12. Tsz F, Lai T, Tim M, Leung Y, Wai E, Chan W, et al. Self-reported reactogenicity of CoronaVac ( Sinovac ) compared with Comirnaty ( Pfizer-BioNTech ): A prospective cohort study with intensive monitoring. Vaccine. 2020;40(January).

13. Qaderi K, Golezar MH, Mardani A, Mallah MA, Moradi B, Kavoussi H, et al. Cutaneous adverse reactions of COVID-19 vaccines: A systematic review. Dermatologic Therapy. 2022;35(5).

14. Cebeci Kahraman F, Savaş Erdoğan S, Aktaş ND, Albayrak H, Türkmen D, Borlu M, et al. Cutaneous reactions after COVID-19 vaccination in Turkey: A multicenter study. Journal of Cosmetic Dermatology. 2022;21(9):3692-703.

15. Leung D, Mu X, Duque JSR, Cheng SMS, Wang M, Zhang W, et al. Safety and immunogenicity of 3 doses of BNT162b2 and CoronaVac in children and adults with inborn errors of immunity. Frontiers in Immunology. 2022;13(September).

16. Can G, Cansu H, Nur S, Inanc I, Karaali R. Waning effectiveness of CoronaVac in real life : A retrospective cohort study in health care workers. Vaccine. 2020;40(January).

17. Al-Ali D, Elshafeey A, Mushannen M, Kawas H, Shafiq A, Mhaimeed N, et al. Cardiovascular and haematological events post COVID-19 vaccination: A systematic review. Journal of Cellular and Molecular Medicine. 2022;26(3):636-53.

18. Ozdede A, Guner S, Ozcifci G, Yurttas B, Toker Dincer Z, Atli Z, et al. Safety of SARS-CoV-2 vaccination in patients with Behcet’s syndrome and familial Mediterranean fever: a cross-sectional comparative study on the effects of M-RNA based and inactivated vaccine. Rheumatology International. 2022;42(6):973-87.

19. Bostan H, Unsal IO, Kizilgul M, Gul U, Sencar ME, Ucan B, et al. Two cases of subacute thyroiditis after different types of SARS-CoV-2 vaccination. Archives of Endocrinology and Metabolism. 2022;66(1):97-103.

20. Miyaji KT, Yuji L, Itto U, Caue L, Caroline A, Sales R, et al. Adverse events following immunization of elderly with COVID-19 inactivated virus vaccine (CoronaVac) in Southeastern Brazil: an active surveillance study. Journal of the Sao Paulo Institute of Tropical Medicine. 2022;64:4-11.

21. Cho K, Park S, Kim EY, Koyanagi A, Jacob L, Yon DK, et al. Immunogenicity of COVID-19 vaccines in patients with diverse health conditions: A comprehensive systematic review. Journal of Medical Virology. 2022;94(9):4144-55.

22. Nantanee R, Aikphaibul P, Jaru-ampornpan P, Sodsai P. Immunogenicity and reactogenicity after booster dose with AZD1222 via intradermal route among adult who had received CoronaVac. Vaccine. 2020;40(January).

23. Kolahchi Z, Khanmirzaei M, Mowla A. Acute ischemic stroke and vaccine-induced immune thrombotic thrombocytopenia post COVID-19 vaccination ; a systematic review. Journal of the Neurological Sciencies. 2022;439(January).

24. Clemens SAC, Weckx L, Clemens R, Mendes AVA, Souza AR, Silveira MB V, et al. Heterologous versus homologous COVID-19 booster vaccination in previous recipients of two doses of CoronaVac COVID-19 vaccine in Brazil (RHH-001): a phase 4, non-inferiority, single blind, randomised study. The Lancet. 2022;6736(15):1-24.

25. Kang W, Shami JJP, Yan VKC, Ye X, Blais JE, Li X, et al. Safety of two-dose COVID-19 vaccination (BNT162b2 and CoronaVac) in adults with cancer: a territory-wide cohort study. Journal of Hematology and Oncology. 2022;15(1):1-6.

26. Lin Z, Cheng M, Zhu F, Yang X, Zuo J, He S. Immunogenicity and safety of different platforms of COVID-19 vaccines given as a third (booster) dose in healthy adults. Journal of Medical Virology. 2022;94(9):4047-52.

27. Gofinet Pasoto S, E. Aikawa N, S.R. Halpern A, K.N. Guedes L, E.B. Villamarín L, A.O. Martins V, et al. Moderate immunogenicity and excellent safety of an inactivated virus vaccine against SARS-CoV-2 in primary Sjögren’s syndrome: a prospective phase 4 controlled trial. 2021;(October):1-11.

28. Bostan H, Kayihan S, Calapkulu M, Hepsen S, Gul U, Ozturk Unsal I, et al. Evaluation of the diagnostic features and clinical course of COVID-19 vaccine–associated subacute thyroiditis. Hormones. 2022;21(3):447-55.

29. Cao C, Qiu F, Lou C, Fang L, Liu F, Zhong J, et al. Safety of inactivated SARS-CoV-2 vaccines in patients with allergic diseases. Respiratory Research. 2022;23(1):1-8.

30. Dundar B, Karahangil K, Elgormus CS, Topsakal HNH. Efficacy of antibody response following the vaccination of SARS-CoV-2 infected and noninfected healthcare workers by two-dose inactive vaccine against COVID-19. Journal of Medical Virology. 2022;94(6):2431-7.

31. Jara A, Undurraga EA, González C, Paredes F, Fontecilla T, Jara G, et al. Effectiveness of an Inactivated SARS-CoV-2 Vaccine in Chile. The New England journal of medicine. septiembre de 2021;385(10):875-84.

32. Netto LC, Ibrahim KY, Picone CM, Alves APPS, Aniceto EV, Santiago MR, et al. Safety and immunogenicity of CoronaVac in people living with HIV: a prospective cohort study. The lancet HIV. mayo de 2022;9(5):e323-31.

33. Cerqueira-Silva T, Katikireddi SV, de Araujo Oliveira V, Flores-Ortiz R, Júnior JB, Paixão ES, et al. Vaccine effectiveness of heterologous CoronaVac plus BNT162b2 in Brazil. Nature medicine. abril de 2022;28(4):838-43.

34. De Souza WM, Muraro SP, Souza GF, Amorim MR, Sesti-Costa R, Mofatto LS, et al. Clusters of SARS-CoV-2 Lineage B.1.1.7 Infection after Vaccination with Adenovirus-Vectored and Inactivated Vaccines. Viruses. 22 de octubre de 2021;13(11):2127.

35. Lu L, Zhang Q, Xiao J, Zhang Y, Peng W, Han X, et al. COVID‐19 vaccine take‐up rate and safety in adults with epilepsy: Data from a multicenter study in China. Epilepsia. enero de 2022;63(1):244-51.

36. Dinc HO, Saltoglu N, Can G, Balkan II, Budak B, Ozbey D, et al. Inactive SARS-CoV-2 vaccine generates high antibody responses in healthcare workers with and without prior infection. Vaccine. enero de 2022;40(1):52-8.

37. Benjamanukul S, Traiyan S, Yorsaeng R, Vichaiwattana P, Sudhinaraset N, Wanlapakorn N, et al. Safety and immunogenicity of inactivated COVID‐19 vaccine in health care workers. Journal of Medical Virology. abril de 2022;94(4):1442-9.

38. Al‐Ali D, Elshafeey A, Mushannen M, Kawas H, Shafiq A, Mhaimeed N, et al. Cardiovascular and haematological events post COVID‐19 vaccination: A systematic review. J Cellular Molecular Medi. febrero de 2022;26(3):636-53.

39. Zeng G, Wu Q, Pan H, Li M, Yang J, Wang L, et al. Immunogenicity and safety of a third dose of CoronaVac, and immune persistence of a two-dose schedule, in healthy adults: interim results from two single-centre, double-blind, randomised, placebo-controlled phase 2 clinical trials. The Lancet Infectious Diseases. abril de 2022;22(4):483-95.

40. Rerknimitr P, Puaratanaarunkon T, Wongtada C, Wittayabusarakam N, Krithin S, Paitoonpong L, et al. Cutaneous adverse reactions from 35,229 doses of Sinovac and AstraZeneca COVID‐19 vaccination: a prospective cohort study in healthcare workers. Acad Dermatol Venereol [Internet]. marzo de 2022 [citado 6 de junio de 2023];36(3). Disponible en: https://onlinelibrary.wiley.com/doi/10.1111/jdv.17761

41. Oskay T, Isık M. Leukocytoclastic vasculitis after the third dose of CoronaVac vaccination. Clin Rheumatol. junio de 2022;41(6):1931-3.

42. Sritipsukho P, Khawcharoenporn T, Siribumrungwong B, Damronglerd P, Suwantarat N, Satdhabudha A, et al. Comparing real-life effectiveness of various COVID-19 vaccine regimens during the delta variant-dominant pandemic: a test-negative case-control study. Emerging Microbes & Infections. 31 de diciembre de 2022;11(1):585-92.

43. Supangat, Sakinah EN, Nugraha MY, Qodar TS, Mulyono BW, Tohari AI. COVID-19 Vaccines Programs: adverse events following immunization (AEFI) among medical Clerkship Student in Jember, Indonesia. BMC Pharmacol Toxicol. diciembre de 2021;22(1):58.

44. Shao W, Chen X, Zheng C, Liu H, Wang G, Zhang B, et al. Effectiveness of COVID-19 vaccines against SARS-CoV-2 variants of concern in real-world: a literature review and meta-analysis. Emerging Microbes & Infections. 31 de diciembre de 2022;11(1):2383-92.

45. Chen F, Zhong Y, Li J, Luo J. Dynamic changes of SARS-CoV-2 specific IgM and IgG among population vaccinated with COVID-19 vaccine. Epidemiol Infect. 2022;150:e74.

46. Lau O, Vadlamudi NK. Immunogenicity and Safety of the COVID-19 Vaccines Compared With Control in Healthy Adults: A Qualitative and Systematic Review. Value in Health. mayo de 2022;25(5):717-30.

47. Mok CKP, Cohen CA, Cheng SMS, Chen C, Kwok K, Yiu K, et al. Comparison of the immunogenicity of BNT162b2 and CoronaVac COVID ‐19 vaccines in Hong Kong. Respirology. abril de 2022;27(4):301-10.

48. Riad A, Sağıroğlu D, Üstün B, Pokorná A, Klugarová J, Attia S, et al. Prevalence and Risk Factors of CoronaVac Side Effects: An Independent Cross-Sectional Study among Healthcare Workers in Turkey. JCM. 15 de junio de 2021;10(12):2629.

49. Chen L, Cai X, Zhao T, Han B, Xie M, Cui J, et al. Safety of Global SARS-CoV-2 Vaccines, a Meta-Analysis. Vaccines. 12 de abril de 2022;10(4):596.

50. Calil VMLT, Palmeira P, Zheng Y, Krebs VLJ, Carvalho WBD, Carneiro-Sampaio M. CoronaVac can induce the production of anti-SARS-CoV-2 IgA antibodies in human milk. Clinics. 2021;76:e3185.

51. Clavero R, Parra-Lucares A, Méndez-Valdés G, Villa E, Bravo K, Mondaca E, et al. Humoral Immune Response of BNT162b2 and CoronaVac Vaccinations in Hemodialysis Patients: A Multicenter Prospective Cohort. Vaccines. 16 de septiembre de 2022;10(9):1542.

52. Kara Z, Akçin R, Demir AN, Dinç HÖ, Taşkın HE, Kocazeybek B, et al. Antibody Response to SARS-CoV-2 Vaccines in People with Severe Obesity. OBES SURG. septiembre de 2022;32(9):2987-93.

53. Chui CSL, Fan M, Wan EYF, Leung MTY, Cheung E, Yan VKC, et al. Thromboembolic events and hemorrhagic stroke after mRNA (BNT162b2) and inactivated (CoronaVac) covid-19 vaccination: A self-controlled case series study. eClinicalMedicine. agosto de 2022;50:101504.

54. Demirhindi H, Mete B, Tanir F, Kara E, Kibar F, Cetiner S, et al. Effect of Heterologous Vaccination Strategy on Humoral Response against COVID-19 with CoronaVac Plus BNT162b2: A Prospective Cohort Study [Internet]. MEDICINE & PHARMACOLOGY; 2022 mar [citado 6 de junio de 2023]. Disponible en: https://www.preprints.org/manuscript/202203.0046/v1

55. Shiravi AA, Ardekani A, Sheikhbahaei E, Heshmat-Ghahdarijani K. Cardiovascular Complications of SARS-CoV-2 Vaccines: An Overview. Cardiol Ther. marzo de 2022;11(1):13-21.

56. Fu Y, Chen F, Cui L, Zhao Y, Zhang H, Fu S, et al. Immunological Analysis of People in Northeast China after SARS-CoV-2 Inactivated Vaccine Injection. Vaccines. 16 de septiembre de 2021;9(9):1028.

57. Pinpathomrat N, Intapiboon P, Seepathomnarong P, Ongarj J, Sophonmanee R, Hengprakop J, et al. Immunogenicity and safety of an intradermal ChAdOx1 nCoV-19 boost in a healthy population. npj Vaccines. 13 de mayo de 2022;7(1):52.

58. Ben Saida I, Maatouk I, Toumi R, Bouslama E, Ben Ismail H, Ben Salem C, et al. Acquired Thrombotic Thrombocytopenic Purpura Following Inactivated COVID-19 Vaccines: Two Case Reports and a Short Literature Review. Vaccines. 24 de junio de 2022;10(7):1012.

59. Chaijaras S, Seree-aphinan C, Rutnin S, Ngamjanyaporn P, Rattanakaemakorn P. Serum sickness-like reaction following an administration of the first dose of inactivated COVID19 vaccine. JAAD Case Reports. enero de 2022;19:21-4.

60. Oliveira CFD, Neto WFF, Silva CPD, Ribeiro ACS, Martins LC, Sousa AWD, et al. Absence of Anti-RBD Antibodies in SARS-COV-2 Infected or Naive Individuals Prior to Vaccination With CoronaVac Leads to Short Protection of Only Four Months Duration [Internet]. BIOLOGY; 2022 mar [citado 6 de junio de 2023]. Disponible en: https://www.preprints.org/manuscript/202203.0101/v1

61. Chan RWY, Liu S, Cheung JY, Tsun JGS, Chan KC, Chan KYY, et al. The Mucosal and Serological Immune Responses to the Novel Coronavirus (SARS-CoV-2) Vaccines. Front Immunol. 12 de octubre de 2021;12:744887.

62. Dadras O, Mehraeen E, Karimi A, M. Tantuoyir M, Afzalian A, Nazarian N, et al. Safety and Adverse Events Related to Inactivated COVID-19 Vaccines and Novavax; a Systematic Review. Archives of Academic Emergency Medicine. 7 de julio de 2022;10(1):e54.

63. -Olivos EN, -Vázquez FJM, Sánchez-Navarro MDR, Díaz-Martínez DA, Gallardo-Luna MDJ, Raygoza NP. Reactivity and Safety of BioNTech/Pfizer® Vaccine Anti-SARS-CoV-2, in Health Personnel from the Mexican State of Guanajuato. Biomed Pharmacol J. 30 de junio de 2022;15(2):993-1003.

64. Sibbel S, McKeon K, Luo J, Wendt K, Walker AG, Kelley T, et al. Real-World Effectiveness and Immunogenicity of BNT162b2 and mRNA-1273 SARS-CoV-2 Vaccines in Patients on Hemodialysis. JASN. enero de 2022;33(1):49-57.

65. Ao L, Lu T, Cao Y, Chen Z, Wang Y, Li Z, et al. Safety and immunogenicity of inactivated SARS-CoV-2 vaccines in people living with HIV. Emerging Microbes & Infections. 31 de diciembre de 2022;11(1):1126-34.

66. Díaz LA, Fuentes-López E, Lazo M, Kamath PS, Arrese M, Arab JP. Vaccination against COVID-19 decreases hospitalizations in patients with cirrhosis: Results from a nationwide analysis. Vol. 42, Liver International. Wiley-Blackwell; 2022. p. 942-4.

67. Choi M, Yu SY, Cheong C, Choe YJ, Choi SH. Efficacy and Safety of COVID-19 Vaccines in Children Aged 5 to 11 Years: A Systematic Review. Pediatric Infection and Vaccine. 2022;29(1):28-36.

68. Bertola G, Bianchi R, Giambona S, Ruiz-Luna R, Martucci F, Berra SA. Three cases of subacute thyroiditis following SARS-CoV-2 Vaccine. Italian Journal of Medicine. 2022;17-17.

69. Ozdede A, Güner S, Ozcifci G, Yurttas B, Toker Dincer Z, Atli Z, et al. POS1255 SAFETY OF THE PFIZER/BIONTECH AND SINOVAC/CORONAVAC VACCINES AMONG PATIENTS WITH BEHCET’S SYNDROME AND FAMILIAL MEDITERRANEAN FEVER. Annals of the Rheumatic Diseases. junio de 2022;81(Suppl 1):962.2-963.

70. Czarnowska A, Tarasiuk J, Zajkowska O, Wnuk M, Marona M, Nowak K, et al. Safety of Vaccines against SARS-CoV-2 among Polish Patients with Multiple Sclerosis Treated with Disease-Modifying Therapies. Vaccines. 1 de mayo de 2022;10(5):763.

71. Chen YJ, Huang WN, Chen HH, Chen HW, Cheng PL, Chen JP, et al. POS1249 EFFICACY AND SAFETY OF ChAdOx1 nCoV-19/AZD1222 AND mRNA-1273 VACCINES: A COMPARATIVE STUDY IN PATIENTS WITH AUTOIMMUNE RHEUMATIC DISEASES. Annals of the Rheumatic Diseases. 1 de junio de 2022;81(Suppl 1):959-959.

72. Cox R, Parish M, Thapa B, McKenna E, Chakrabarti S. Safety of vaccines against coronavirus disease 2019 (COVID-19) in patients receiving systemic therapy for solid tumors. JACCP Journal of the American College of Clinical Pharmacy. diciembre de 2021;4(12):1704-1704.

73. Hasseli R, Hoyer BF, Lorenz HM, Pfeil A, Richter J, Regierer A, et al. Safety of COVID-19 vaccines after first vaccination in patients with rheumatic diseases in a patient reported survey. En: Arthritis and Rheumatology. 2021. p. 193-5.

74. Murali S, Sakthivel M, Pattabi K, Venkatasamy V, Thangaraj JWV, Shete A, et al. Effectiveness of the ChAdOx1 nCoV-19 Coronavirus Vaccine (Covishield^TM^) in Preventing SARS-CoV2 Infection, Chennai, Tamil Nadu, India, 2021. Vaccines. 1 de junio de 2022;10(6):970.

75. Ortiz-Prado E, Izquierdo-Condoy JS, Fernandez-Naranjo R, Simbaña-Rivera K, Vásconez-González J, Lincango Naranjo EP, et al. A Comparative Analysis of a Self-Reported Adverse Events Analysis after Receiving One of the Available SARS-CoV-2 Vaccine Schemes in Ecuador. Vaccines. 1 de julio de 2022;10(7).

76. Cheng H, Peng Z, Luo W, Si S, Mo M, Zhou H, et al. Efficacy and safety of covid-19 vaccines in phase iii trials: A meta-analysis. Vaccines. 1 de junio de 2021;9(6).

77. Lv M, Luo X, Shen Q, Lei R, Liu X, Liu E, et al. Safety, immunogenicity, and efficacy of COVID-19 vaccines in children and adolescents: A systematic review. Vol. 9, Vaccines. Vaccines (Basel); 2021.

78. Cheng H, Peng Z, Si S, Alifu X, Zhou H, Chi P, et al. Immunogenicity and Safety of Homologous and Heterologous Prime–Boost Immunization with COVID-19 Vaccine: Systematic Review and Meta-Analysis. Vaccines. 1 de mayo de 2022;10(5):798.

79. Chua SKK, Soh QY, Saffari SE, Tan EK. Prognosis of Guillain–Barré Syndrome Linked to COVID-19 Vaccination. Brain Sciences. 1 de junio de 2022;12(6).

80. Chantasrisawad N, Puthanakit T, Kornsitthikul K, Jaru-Ampornpan P, Tawan M, Matapituk P, et al. Immunogenicity to SARS-CoV-2 Omicron variant among school-aged children with 2-dose of inactivated SARS-CoV-2 vaccines followed by BNT162b2 booster. Vaccine: X. 1 de diciembre de 2022;12.

81. Kanokudom S, Assawakosri S, Suntronwong N, Auphimai C, Nilyanimit P, Vichaiwattana P, et al. Safety and Immunogenicity of the Third Booster Dose with Inactivated, Viral Vector, and mRNA COVID-19 Vaccines in Fully Immunized Healthy Adults with Inactivated Vaccine. Vaccines. 1 de enero de 2022;10(1).

82. Abarca K, Iturriaga C, Urzúa M, Le Corre N, Pineda A, Fernández C, et al. Safety and Non-Inferiority Evaluation of Two Immunization Schedules with an Inactivated SARS-CoV-2 Vaccine in Adults: A Randomized Clinical Trial. Vaccines. 1 de julio de 2022;10(7):1082.

83. Boongird S, Setthaudom C, Kitpermkiat R, Prasongtanakij S, Srisala S, Chuengsaman P, et al. Durability of Humoral and Cellular Immunity after an Extended Primary Series with Heterologous Inactivated SARS-CoV-2 Prime-Boost and ChAdOx1 nCoV-19 in Dialysis Patients (ICON3). Vaccines. 1 de julio de 2022;10(7):1064.

84. Kang M, Yi Y, Li Y, Sun L, Deng A, Hu T, et al. Effectiveness of Inactivated COVID-19 Vaccines against Illness Caused by the B.1.617.2 (Delta) Variant during an Outbreak in Guangdong, China. Annals of Internal Medicine. 1 de abril de 2022;175(4):533-40.

85. Cheng SS, Mok CK, Li JK, Ng SS, Lam BH, Jeevan T, et al. Plaque-neutralizing antibody to BA.2.12.1, BA.4 and BA.5 in individuals with three doses of BioNTech or CoronaVac vaccines, natural infection and breakthrough infection. Journal of Clinical Virology. 1 de noviembre de 2022;156.

86. Shenyu W, Xiaoqian D, Bo C, Xuan D, Zeng W, Hangjie Z, et al. Immunogenicity and safety of a SARS-CoV-2 inactivated vaccine (CoronaVac) co-administered with an inactivated quadrivalent influenza vaccine: A randomized, open-label, controlled study in healthy adults aged 18 to 59 years in China. Vaccine. 26 de agosto de 2022;40(36):5356-65.

87. Li JX, Wu SP, Guo XL, Tang R, Huang BY, Chen XQ, et al. Safety and immunogenicity of heterologous boost immunisation with an orally administered aerosolised Ad5-nCoV after two-dose priming with an inactivated SARS-CoV-2 vaccine in Chinese adults: a randomised, open-label, single-centre trial. The Lancet Respiratory Medicine. 1 de agosto de 2022;10(8):739-48.

88. Demir E, Dheir H, Safak S, Serra Artan A, Sipahi S, Turkmen A. Differences in clinical outcomes of COVID-19 among vaccinated and unvaccinated kidney transplant recipients. Vaccine. 26 de mayo de 2022;40(24):3313-9.

89. Dheir H, Tocoglu A, Toptan H, Pinar M, Demirci T, Koroglu M, et al. Short and mid-term SARS-CoV-2 antibody response after inactivated COVID-19 vaccine in hemodialysis and kidney transplant patients. Journal of Medical Virology. 1 de julio de 2022;94(7):3176-83.

90. Cheng FWT, Fan M, Wong CKH, Chui CSL, Lai FTT, Li X, et al. The effectiveness and safety of mRNA (BNT162b2) and inactivated (CoronaVac) COVID-19 vaccines among individuals with chronic kidney diseases. Kidney International. 2022;102(4).

91. Camacho Moll ME, Salinas Martínez AM, Tovar Cisneros B, García Onofre JI, Navarrete Floriano G, Bermúdez de León M. Extension and Severity of Self-Reported Side Effects of Seven COVID-19 Vaccines in Mexican Population. Frontiers in Public Health. 14 de marzo de 2022;10:387.

92. Nguyen B, Lalama MJ, Gamret AC, Elman SA. Cutaneous symptoms of connective tissue diseases after COVID-19 vaccination: a systematic review. Vol. 61, International Journal of Dermatology. John Wiley & Sons, Ltd; 2022. p. e238-41.

93. Cucunawangsih C, Wijaya RS, Lugito NPH, Suriapranata I. Antibody response after a third dose mRNA-1273 vaccine among vaccinated healthcare workers with two doses of inactivated SARS-CoV-2 vaccine. International Journal of Infectious Diseases. 1 de mayo de 2022;118:116-8.
